# Age‐specific reproduction in female Steller sea lions in Southeast Alaska

**DOI:** 10.1002/ece3.10515

**Published:** 2023-09-27

**Authors:** Kelly K. Hastings, Lauri A. Jemison, Grey W. Pendleton, Devin S. Johnson, Thomas S. Gelatt

**Affiliations:** ^1^ Alaska Department of Fish and Game Juneau Alaska USA; ^2^ Protected Resources Division, National Marine Fisheries Service Pacific Islands Fisheries Science Center Honolulu Hawaii USA; ^3^ Marine Mammal Laboratory, National Marine Fisheries Service Alaska Fisheries Science Center Seattle Washington USA

**Keywords:** life history, marine heatwave, marine mammal, natality, otariid, pinniped, population dynamics, population models, reproduction, reproductive rate

## Abstract

Age‐, region‐, and year‐specific estimates of reproduction are needed for monitoring wildlife populations during periods of ecosystem change. Population dynamics of Steller sea lions (*Eumetopias jubatus*) in Southeast Alaska varied regionally (with high population growth and survival in the north vs. the south) and annually (with reduced adult female survival observed following a severe marine heatwave event), but reproductive performance is currently unknown. We used mark‐resighting data from 1006 Steller sea lion females marked as pups at ~3 weeks of age from 1994 to 1995 and from 2001 to 2005 and resighted from 2002 to 2019 (to a maximum age of 25) to examine age‐, region‐, and year‐specific reproduction. In the north versus the south, age of first reproduction was earlier (beginning at age 4 vs. age 5, respectively) but annual birth probabilities of parous females were reduced by 0.05. In an average year pre‐heatwave, the proportion of females with pup at the end of the pupping season peaked at ages 12–13 with ~0.60/0.65 (north/south) with pup, ~0.30/0.25 with juvenile, and ~0.10 (both regions) without a dependent. In both regions, reproductive senescence was gradual after age 12: ~0.40, 0.40, and 0.20 of females were in these reproductive states, respectively, by age 20. Correcting for neonatal mortality, true birth probabilities at peak ages were 0.66/0.72 (north/south). No cost of reproduction on female survival was detected, but pup production remained lower (−0.06) after the heatwave event, which if sustained could result in population decline in the south. Reduced pup production and greater retention of juveniles during periods of poor prey conditions may be an important strategy for Steller sea lions in Southeast Alaska, where fine‐tuning reproduction based on nutritional status may improve the lifetime probability of producing pups under good conditions in a variable and less productive environment.

## Graphical abstract

We used mark‐resighting data from 1006 Steller sea lion females marked as pups at age ~3 weeks and resighted to age 25 in Southeast Alaska to examine costs of reproduction and age‐, region‐, and year‐specific reproductive patterns. After first reproduction at ages 4–7, the proportion of females with a pup at the end of the pupping season peaked at ages 12–13 with ~0.60 to 0.65 of females with a pup, ~0.25 to 0.30 with a juvenile, and ~0.10 without a dependent. In this study, we report new regional, annual and age‐specific patterns in Steller sea lion reproduction including (1) earlier ages of first reproduction related to high environmental productivity, (2) reduced natality following severe marine heatwave, and (3) age‐specific reproductive senescence.

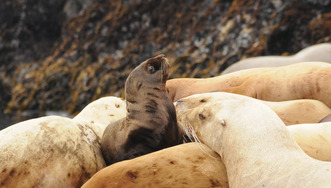

## 1 INTRODUCTION

Age‐structured survival and reproduction determine both individual fitness (Bouwhuis et al., 2012) and changes in population abundance and are therefore key demographic processes for monitoring natural populations (Eberhardt, 1985). In large mammal species characterized by low mass‐specific metabolic rates and intrinsic rates of increase (Hennemann, 1983), population change is most sensitive to changes in adult survival probabilities (Gaillard et al., 2000). However, changes in birth probabilities may drive population change during the initial phases of growth (Albon et al., 2000), in increasing populations (Coulson & Hudson, 2003) and when reproduction is more annually variable and environmentally sensitive than adult survival (Coulson et al., 2005; Manlik et al., 2016 and references therein). Therefore, monitoring reproduction is critical for large mammal species, including annual and age‐specific variability (e.g., recruitment and senescence) and covariance between survival and reproduction, which may alter outcomes of population models (Colchero et al., 2019; Doak et al., 2005).

The Steller sea lion, *Eumetopias jubatus*, an eared seal species of the family Otariidae, is an important top predator in the North Pacific Ocean occurring from California around the Pacific Rim to Alaska, Russia and Japan (King, 1983). Population declines of up to 80% from the 1970s to 2003 (Fritz et al., 2016) resulted in the listing of the species throughout much of its' range under the U.S. Endangered Species Act. Recent estimates of age‐specific survival probabilities (Altukhov et al., 2015; Fritz et al., 2014; Hastings et al., 2011; Maniscalco, 2014; Warlick et al., 2022; Wright et al., 2017) are useful for population viability models, but age‐specific information on reproduction is sparse. Models have relied on reproductive rate estimates from the 1970s and 1980s which were based on pregnancy rates of cross‐sectional samples (Pitcher & Calkins, 1981; Pitcher et al., 1998) that assumed no reproductive senescence (York, 1994). Reproductive senescence is expected in nearly all mammals (Comizzoli & Ottinger, 2021) but remains unstudied in Steller sea lions. Birth probabilities are best provided by direct observations of known individual females during the pupping season due to high rates of late‐term abortions (Pitcher & Calkins, 1981); longitudinal sampling of marked known‐aged females provides ideal information (Le Boeuf et al., 2019). However, robust estimates of age‐specific reproduction are available for less than half of the 15 extant otariid species (Childerhouse et al., 2010; Dabin et al., 2004; Kalberer et al., 2018; Lunn et al., 1994; McKenzie et al., 2007; Melin et al., 2012).

**FIGURE 1:**
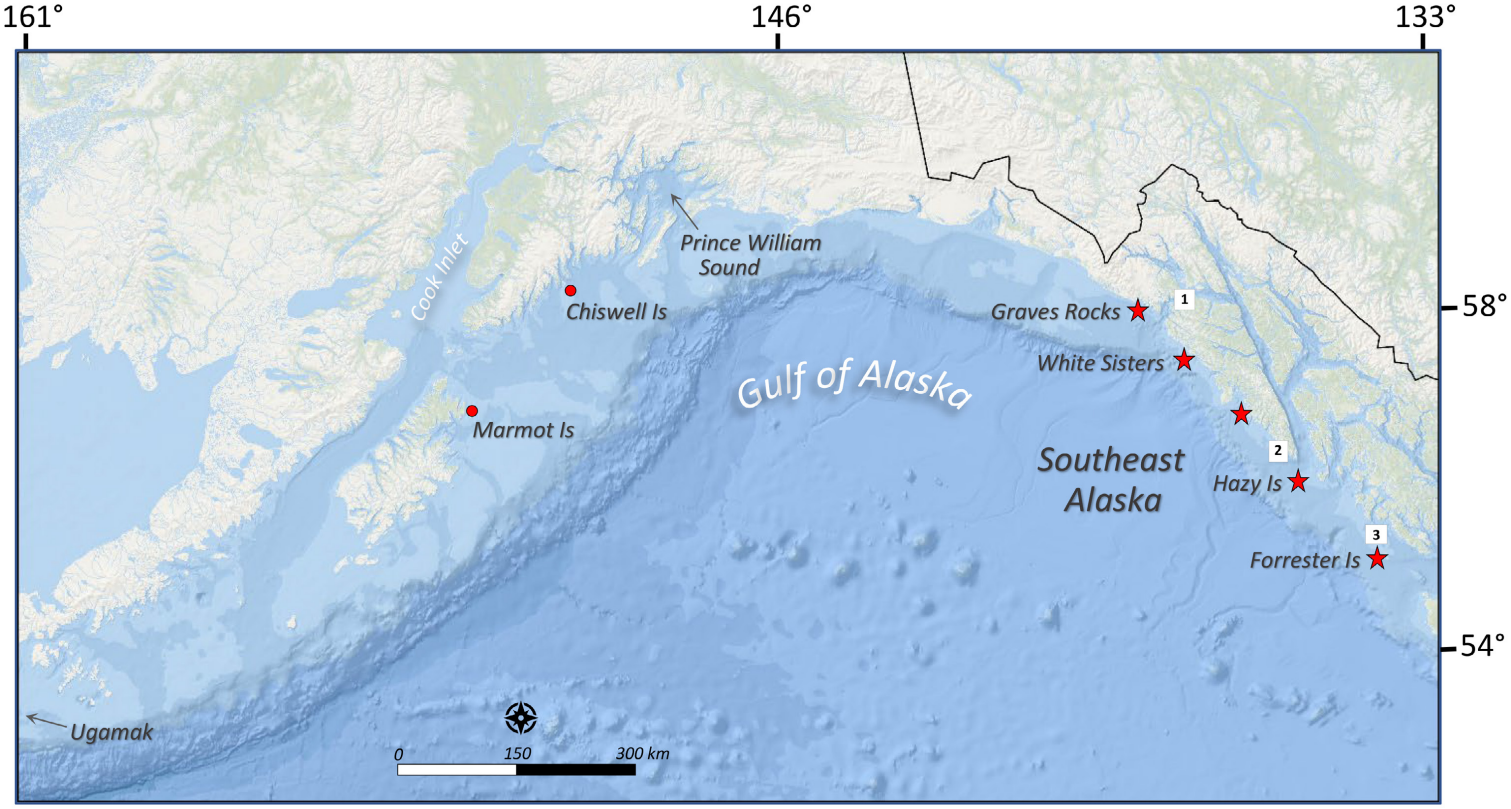
Map of Steller sea lion rookeries in the Gulf of Alaska. Four rookeries (red stars) where pups were marked in Southeast Alaska were: Forrester Islands (south region), Hazy Islands (south region), White Sisters (north region), and Graves Rocks (north region); no pups were marked at a fifth small rookery Biali Rocks. Boxed = 1: Inian Islands, 2: Sea Lion Rocks Puffin Bay, and 3: Wolf Rock.

In addition to age effects, regional and annual shifts in reproduction may indicate reproductive strategies females use to cope with environmental variation, a primary concern for Steller sea lion conservation (NMFS, 2008). Population dynamics vary regionally and annually for Steller sea lions in Southeast Alaska. Regional differences suggest a more productive environment and/or reduced density dependence in the north (rookeries White Sisters and Graves Rocks) versus the south (rookeries Hazy and Forrester Islands; Figure 1) with high population growth, smaller population size, more restricted animal movements, larger neonates and high juvenile survival in the north, compared to population stability, large population size, smaller neonates, lower juvenile survival, higher survival cost of weaning for juveniles, and more extensive animal movements in the south, where the population is considered near carrying capacity (Hastings et al., 2011, 2021; Jemison et al., 2018; Mathews et al., 2011; Pitcher et al., 2007). Therefore, regional differences in reproductive output may indicate female response to variation in environmental productivity and/or local abundance.

Annual variation in population dynamics may also indicate sea lion response to abrupt environmental change: an abrupt decline of −0.05 to −0.23 in adult female Steller sea lion survival occurred in Southeast Alaska, in Prince William Sound and at Chiswell Island (Figure 1) during and following the severe North Pacific marine heatwave of 2014–2016 (PMH; Hastings et al., 2023). The effects of the PMH on reproduction are of interest because widescale and persistent changes in the Gulf of Alaska food web during the PMH are well documented (Arimitsu et al., 2021; Suryan et al., 2021), and food intake relative to whole‐body energy balance strongly determines reproductive success in female mammals (Bronson, 1985; Wade & Schneider, 1992), including Steller sea lions (Pitcher et al., 1998). Whether reproductive state contributed to the high female mortality observed during the PMH is also of interest. The important trade‐off between female survival and fecundity (Stearns, 1989) has not yet been documented for Steller sea lions (Maniscalco et al., 2014). Both reduced (due to energy costs of raising young) and increased (due to higher survival and reproduction in higher quality individuals) female survival have been associated with offspring production in other otariids (Beauplet et al., 2006; Boyd et al., 1995). Marine heatwaves are predicted to increase in frequency and severity with ocean warming (Oliver et al., 2018), and therefore current vital rate information is particularly needed for models addressing the effects of climate change on Steller sea lions and other marine mammal populations (Albouy et al., 2020).

Estimating otariid reproductive rates with mark‐recapture studies may be challenging due to the need to estimate both pup production and juvenile retention (multiple reproductive states) with imperfect state detection. Otariids produce a single pup at a time (twinning is rare) and lactation is energetically demanding: females must feed during lactation (for at least 9–12 months in all but two species) to sustain both their own and their offspring's growth and survival (Bonner, 1984) which also requires them to remain nearshore ~year‐round (Costa & Valenzuela‐Toro, 2021). In nearly half of otariid species, including Steller sea lions, females may retain dependent juveniles for >1 year in lieu of new pup production (Hastings et al., 2021). Multiple potential reproductive states complicate studies of reproduction and associated statistical models because demographic and observational processes are often state dependent. Often, the detection of reproductive state is imperfect because when females are observed, they are not always physically associated with their offspring, and some states may not be definitively observed (e.g., nonbreeder or weaned; Hastings et al., 2021; Johnson et al., 2016). For statistical models to accurately estimate reproduction, female resighting and state detection probabilities must also be estimated. Modeling only two states (with and without pup) will not yield accurate estimates of reproduction when behavior varies significantly for females with juveniles versus those without a dependent.

In this study, we used appropriate models to address these complexities for mark‐resighting data from 1006 Steller sea lion females marked as pups at 3 weeks of age at their natal rookeries in the northern and southern regions of Southeast Alaska (from 1994 to 1995 and from 2001 to 2005) and resighted from 2002 to 2019 to a maximum age of 25 years. Here, we estimate age‐specific reproductive performance, and regional and year variation in reproductive performance, with particular interest in effects of the PMH on reproductive output, including an evaluation of the cost of reproduction to female survival.

## 2 MATERIALS AND METHODS

### 2.1 Field data collection

Steller sea lions produce pups from late May through early July (Pitcher et al., 2001). In the Gulf of Alaska, ~80% of pups are born within 20–25 days and on average, 98% of pups are produced by 28–29 June (Edie, 1977; Kuhn et al., 2017; Sandegren, 1970, Report S1 in Hastings et al., 2018). At the end of the pupping season (average date was 28 June, ranging 24 June–3 July), 2‐ to 4‐week‐old Steller sea lion female pups were hot‐branded at four out of the five rookeries that exist in Southeast Alaska (1994–1995 [*n* = 116] and 2001–2005 [*n* = 890]; Table 1, Figure 1). Pups were not marked at one small (<100 pups) rookery, Biali Rocks (Figure 1).

**TABLE 1:** Number of female Steller sea lion pups marked in Southeast Alaska, 1994–2005, by natal rookery and year.

Natal rookery	1994	1995	2001	2002	2003	2004	2005	Total
Forrester	187^a^	185^a^	138	72	112	125		819
Hazy			99		43		109	251
White Sisters				58		40	58	156
Graves				17			19	36
Total	187	185	237	147	155	165	186	1262

^a^ Although 187 and 185 female pups were marked in 1994 and 1995, only 56 and 60, respectively, were included in this study (those seen at least one time >2004, after reproductive rate surveys began in 2005; for a total number of females included in statistical models of 1006). See Figure 1 for natal rookeries.

During each marking session, a workable area on the rookery was chosen, usually containing 75–200 pups. All pups in the area were carefully corralled, monitored, and sampled by a large field crew (Hastings et al., 2009). All pups >20 kg were marked to ensure a representative sample; pups <20 kg were not branded but received a dye mark on their fur and/or a flipper tag. By marking at the end of the pupping season, few pups (<5%) were of insufficient weight and were likely the latest‐born pups. We assume this method of obtaining a marked sample of Steller sea lion pups yielded a representative sample. Methods of animal handling, marking, and observation were approved by the Alaska Department of Fish and Game (ADFG) Institutional Animal Care and Use Committee and under permits issued by the US National Marine Fisheries Service (NMFS) to the ADFG. Branding has been used extensively as a method for permanent marking of pinnipeds, and several studies have reported a lack of effect on survival or animal health using this method (Hastings et al., 2009; McMahon et al., 2006; Merrick et al., 1996). This method was particularly required for Steller sea lions in Alaska for which very high tag loss rates for animals marked as pups and poor visibility of tags leading to insufficient resighting rates precluded the collection of vital rate information (Hastings et al., 2017; Merrick et al., 1996).

Resighting surveys of marked animals occurred at all rookeries and major haulouts in Southeast Alaska during dedicated large‐scale boat‐based surveys and one field camp each summer from 2002 to 2019 (Hastings et al., 2011; Pendleton et al., 2006). Other resighting data were collected each summer throughout the Gulf of Alaska and from sites ranging from California to Russia and into the Bering Strait by multiple agencies and individuals, allowing full coverage of the geographic range and preventing biases in estimated parameters due to emigration.

Surveys specifically to address reproductive status of females were formalized in 2005 with the potential recruitment (i.e., first production of a pup in an individuals' lifetime) of the new cohorts at age 4. Reproductive surveys included 1–2 half‐day surveys over 3–4 consecutive days; multiple, standardized, independent surveys within the survey window per year at each rookery were conducted to allow the probability of detecting offspring and females based on reproductive state to be estimated precisely. During reproductive surveys, we attempted to observe each marked female 3–4 times for at least 30–40 min total, formally recording reproductive status every ~10 min based on specific behaviors observed (Hastings et al., 2021). For this analysis, females were considered to have a dependent on an occasion if the dependent was seen suckling from (83.9% of 1306 with pup sightings), laying on top of (10.6%), or reuniting with the female (5.5%; Hastings et al., 2021). Other less‐definitive behaviors (e.g., brief physical interactions, female and pup lying next to each other) were not considered to be sufficient to classify a female as having a dependent offspring.

Reproductive surveys were conducted in late June–mid July each year to ensure nearly all pups had been born and also to coincide with the time when pups were originally marked to provide complementary survival and reproductive information for population models. For example, the first year survival interval was from ~3 weeks of age until 1 year of age (i.e., excluded the period of higher neonatal mortality during the first 3 weeks of life; Hastings, 2017). Reproductive status in late June–mid July included neonatal mortality (i.e., was true birth probability minus neonatal mortality) to provide the best complimentary information. Ideally, observations throughout the pupping season at all rookeries would be used to estimate true birth probabilities independently from neonatal mortality, but such observations have been conducted in recent decades at only four rookeries in the Gulf of Alaska: Forrester, Marmot, Ugamak, and Chiswell Islands (Figure 1). Our survey protocol was the next best option to provide widescale coverage of rookeries that could not all be observed daily throughout the pupping season. Failure to include early pup mortality (~0.20 from birth to 3 weeks) in first‐year survival estimates (0.57 from age 3 weeks to 1 year, Hastings et al., 2011) produced significant overestimates of population trend for the Forrester Island rookery (Hastings, 2017).

### 2.2 Capture histories and mark‐recapture statistical modeling

Data included in our study were all photograph‐confirmed resightings (to prevent identification errors) of females marked between 2001 and 2005 in Southeast Alaska, all of which entered the dataset as Prebreeders at age 0 (*n* = 890 for estimates of age of first reproduction). Females marked at Forrester Island from 1994 to 1995 (Pendleton et al., 2006) and also seen ≥2005 (when dedicated reproductive rate surveys were initiated) were also included, and their age at first entry into the dataset was specified (*n* = 56 and 60 born in 1994 and 1995, respectively, for a total of 1006 females in the dataset). These 116 females did not contribute to the estimates of age of first reproduction but only to reproductive probabilities of parous females. Ages possible in our data were 24–25 years for the 1994–1995 cohorts, and 14–18 years for the 2001–2005 cohorts. Because marking occurred only from 2001 to 2005 for the new cohorts, age of first reproduction was based primarily on the early years of this study (~2005 to 2012) whereas reproductive rates of parous females spanned all possible ages and years. After winter 2013 (when the PMH began), ages of females ranged 19–25 for the old cohorts and 9–18 for the new cohorts.

Capture histories were created from these resighting data to allow transitions between reproductive states and also movement between rookeries and haulouts to be estimated, because we expected that probability of resighting females of different reproductive states varied between rookeries and haulouts. Capture histories were created for each female with a single occasion per year from 2001 to 2004 and four occasions per year from 2005 to 2019 (63 occasions in total). From 2001 to 2004, sightings from May to August were condensed to a single sighting per year. Starting in 2005 with the initiation of reproductive surveys, the four occasions per year included 3 days of sightings at rookeries (several sightings in a day at a rookery were summarized as a single sighting per day as within‐day observations were not independent; Hastings et al., 2021), followed by one occasion which summarized any sighting at a haulout during the survey period that year. Therefore, time scales differed for occasion types: probabilities were for daily surveys for rookery occasions and were per summer survey for haulout occasions. Movement probabilities between rookeries and haulouts were addressed through the structure of the capture histories (as separate time occasions coupled with a code), whereas reproductive state was formally treated as a multinomial state in the capture history (see Appendix 1 for capture history example).

Four reproductive states were possible: (1) Prebreeder (nulliparous), (2) With‐Pup, (3) With‐Juvenile, and (4) No‐Dependent (parous). With‐Pup and With‐Juvenile were observable states; Prebreeder and No‐Dependent were unobservable (i.e., could not be determined based on observation). On each occasion, sightings of females were coded as “0” if not seen, “u” if seen but reproductive state was uncertain, “B” if seen as With‐Pup, and “J” if seen as With‐Juvenile. The state “Prebreeder” (“P”) occurred once per capture history on the initial release only, which was at age 0 for the 2001–2005 cohorts (Appendix 1). As described earlier, the 116 females from the 1994 and 1995 cohorts were not necessarily Prebreeders when first observed, but their first nonzero record in their capture histories was coded as such, and these females were treated separately when estimating recruitment probabilities, which instead accounted for the transitioning of these females into the population of knowable state after 2004. Fewer females were observed definitively With‐Juvenile than With‐Pup (211 vs. 902 female*year sightings; Appendix 2). Few Steller sea lion females (1.9%–3.7%) can have both a dependent juvenile and pup during the pupping season (aka “triad”), in which case most often the juvenile is favored over the new pup by the end of the pupping season (Hastings et al., 2021; Maniscalco & Parker, 2009). Of 211 females With‐Juvenile*year, only four (1.9%) were also observed with a pup during the survey window, and for these four cases, the pup data were replaced with the juvenile sightings.

Using these capture histories, we fitted multivariate state Cormack–Jolly–Seber models that allowed imperfect state detection and that were formulated as a hidden Markov process, such that maximum likelihood could be used for parameter estimation (Johnson et al., 2016; Laake et al., 2014). We used the R package *marked* (model “mvmscjs”; Laake et al., 2013; R Core Team, 2022) to estimate parameters and select models based on AIC (Burnham & Anderson, 2002). The models included five parameter types, three of which were nuisance parameters (i.e., of no biological interest but necessary for appropriate modeling of the data). They were (1) female resighting probabilities, (2) conditional probability of detecting the reproductive state (i.e., offspring) given the mother was detected, and (3) movement probabilities between haulouts and rookeries. The two parameters of scientific interest were (4) probabilities of changing reproductive states between years (by age, natal region, and year), and (5) female survival probabilities (in relation to reproductive state and year). Our capture history structure required fixing some parameters, similar to a robust design (closed population methods are used for secondary occasions within a primary period and open population methods are used between primary periods; Pollock, 1982). After 2004, survival probabilities and reproductive state transitions were estimated for the intervals between the fourth (i.e., last) occasion in a year to the first occasion of the next year. Survival was fixed to 1 and reproductive state transitions to 0 between the four occasions within a year starting in 2005. Movement from a rookery to a haulout was possible (due to our capture history structure) only between the third and fourth occasions in a year and from a haulout to a rookery between the fourth occasion in a year and the first occasion in the next year (Appendix 1). Including three separate daily surveys at rookeries prevented bias in estimates of female resighting and offspring detection probabilities that may have resulted from summarizing multiple observations when the number of observations per animal per occasion varied (Hastings et al., 2021).

We used a time‐varying covariate for rookery occasions where “0” indicated “not seen before that year at a rookery” and “1” indicated “seen before that year at a rookery” (variable “sb”, possible in the second and third rookery occasions, Appendix 1). This was included to allow female resighting probability to vary for the first versus subsequent resightings at a rookery within a year, which we suspected varied with reproductive state (e.g., females with no dependent may be more likely to be seen only once, and females with pup may be more likely to be seen again after their first sighting). Six reproductive state transitions could be estimated: Prebreeder:With‐Pup (from Prebreeder in year *x* to With‐Pup in year *x* + 1), With‐Pup:No‐Dependent, With Pup:With‐Juvenile, With‐Juvenile:With‐Pup, With‐Juvenile:No‐Dependent, and No‐Dependent:With‐Pup. The probability of remaining in the same state (Prebreeder:Prebreeder, With‐Pup:With‐Pup, With‐Juvenile:With‐Juvenile, No‐Dependent:No‐Dependent) was estimated as the difference of the other row‐wise probabilities because multinomial variables must sum to 1 (Appendix 3). The probabilities of making impossible reproductive state transitions (shown in gray boxes in Appendix 3) were fixed to 0.

We modeled parameters sequentially (beginning with the global or most complex model for all parameters and then simplifying): first nuisance parameters: female resighting probability, then offspring detection probability, then movement transitions, then parameters of biological interest: reproductive state transitions, and finally female survival probability in relation to reproductive state. Following the hypothesis testing framework of Lebreton et al. (1992), we modeled nuisance parameters first to improve precision of estimates and focus our analyses on our primary parameters of interest (and factors affecting them): reproductive state transitions and female survival probability. Models with fewer parameters and the lowest AIC were considered to be the most‐supported models, particularly when ΔAIC was >3.0 (Burnham & Anderson, 2002).

#### 2.2.1 Nuisance parameters

For female resighting probability, our global model included effects of group*natal rookery (nr) with the effect of “seen before” at a rookery (sb) varying among groups, and year effects (yr) that differed among haulouts (H) and rookeries (R) [nr*group + H:yr + R:yr + sb:group]. Eight groups were: at rookeries—(1) juvenile females aged 0–3, (2) With‐pup females, (3) With‐Juvenile females, (4) No‐Dependent females, and (5) Prebreeders aged 4+; and at haulouts—(6) juvenile females aged 0–3, (7) Prebreeders aged 4+ or No‐Dependent females, and (8) With‐Pup or With‐Juvenile females. Natal rookery effects were fit only for groups at rookeries. We fit 17 additional models. For offspring detection probability, our global model included separate estimates for With‐Pup and With‐Juvenile at each natal rookery group (nr2) [nr2:B + nr2:J] where nr2 was Forrester, Hazy, and White Sisters/Graves Rocks pooled (Figure 1); we fit four additional models. For movement transitions between haulouts (H) and rookeries (R), our global model was [HtoR:togroup_year *x* + 1_ + RtoH:group_year *x*_], where group was five groups (Juveniles aged 0–3/Adult Prebreeders 4+/With‐Pup/With‐Juvenile/No‐Dependent); we fit three simpler models by simplifying group.

Models for movement transition parameters were influenced by observed patterns. Only one marked female was observed with a pup at a haulout (W330 at South Marble Island in 2010); few pups were produced at haulouts in Southeast Alaska (from 2010 to 2019: average of 0.6% of pups were at haulouts during aerial surveys, or 39 at haulouts vs. 6504 at rookeries; Alaska Fisheries Science Center, 2023). Only three females were observed with pup at a rookery and also seen at a nearby haulout in the same survey year (Graves Rocks and Inian Islands: ~30 km distant, Hazy Island and Sea Lion Rocks Puffin Bay: ~45 km, and Lowrie Island and Wolf Rock: ~20 km; Figure 1). Therefore, we expected nearly all pupping occurred at rookeries, and that females with pups at rookeries only very rarely were observed at haulouts during the same survey year.

#### 2.2.2 Parameters of biological interest

For reproductive state transitions, our global model was [Recruit:old + Recruit:new:region:age 3/4/5p + With‐Pup:With‐Juvenile + With‐Pup:No‐Dependent + With‐Juvenile:With‐Pup + With‐Juvenile:No‐Dependent + No‐Dependent:With‐Pup], where region was natal region (north/south; Figure 1), old was the 1994–1995 cohorts and new was the 2001–2005 cohorts, 3/4/5p was age 3/age 4/ages 5+, and Recruit was the reproductive state transition Prebreeder:With‐Pup. First, Recruit:new was simplified by age and region. Then all combinations of age and region effects (as linear, quadratic or basis spline trend with age) were then sequentially added to each of the remaining five reproductive state transitions. Lastly, year and cohort effects were included once region and age effects were accounted for (52 additional models fit).

For female survival probability, our global model was based on previous analyses of these data: ac + nr3 + y1415 + y16 (Hastings et al., 2023), where ac was the annual survival of six age‐classes (age 0, age 1, age 2, age 3–15, age 16–17, and age 18+), natal rookery group (nr3) were three categories (Forrester and Hazy pooled, White Sisters, and Graves Rocks; Figure 1), and y1415/y16 were three poor years for survival of prime‐aged females (2014–2016; Hastings et al., 2023). We fit 25 additional models including models in which survival was affected by reproductive state, by reproductive state*age or *year (the three poor years of survival 2014–2016 and 2014+, the years during and following the PMH).

### 2.3 Derived parameters: Proportions of females alive by reproductive state, corrections for early pup mortality, and resulting estimates of population trend

Derived parameters calculated as functions of estimated reproductive state transitions from the best model included the proportion of the female population alive at each age *i* that were of reproductive states Prebreeder (P_*i*_), With‐Pup (B_*i*_), With‐Juvenile (J_*i*_), and No‐Dependent (N_*i*_). Given female survival probabilities did not vary with reproductive state (see Results 3), estimates of these proportions for the first age possible for each reproductive state were calculated following equations: 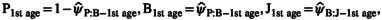 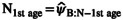. Proportions for subsequent ages *i* were calculated following equations:

$$P_{i}=P_{i-1}*{\hat{\psi }}_{P:P,i-1\;\text{to}\;i},$$

$$\begin{array}{c} B_{i}=\left(P_{i-1}*{\hat{\psi }}_{P:B,i-1\;\text{to}\;i}\right)+\left(B_{i-1}*{\hat{\psi }}_{B:B,i-1\;\text{to}\;i}\right)+\left(J_{i-1}*{\hat{\psi }}_{J:B,i-1\;\text{to}\;i}\right)\\ +\left(N_{i-1}*{\hat{\psi }}_{N:B,i-1\;\text{to}\;i}\right), \end{array}$$

$$J_{i}=\left(B_{i-1}*{\hat{\psi }}_{B:J,i-1\;\text{to}\;i}\right)+\left(J_{i-1}*{\hat{\psi }}_{J:J,i-1\;\text{to}\;i}\right),$$

$$N_{i}=\left(B_{i-1}*{\hat{\psi }}_{B:N,i-1\;\text{to}\;i}\right)+\left(J_{i-1}*{\hat{\psi }}_{J:N,i-1\;\text{to}\;i}\right)+\left(N_{i-1}*{\hat{\psi }}_{N:N,i-1\;\text{to}\;i}\right),$$

where $\hat{\psi }$ was the estimated probability of changing reproductive states between years (e.g., $\hat{\psi }$_P:B,*i* − 1 to *i*_ was the estimated probability of transitioning from Prebreeder at age *i* − 1 to With‐Pup at age *i* or Prebreeder:With‐Pup). Confidence intervals (95% CI) for derived values were approximated using a multivariate normal parametric bootstrap with the mean equal to the maximum likelihood estimate and the covariance matrix equal to the negative Hessian of the log‐likelihood function, following Johnson et al. (2016).

To correct the estimates of proportion with pup (B_*i*_) for early pup mortality to 3 weeks of age (to provide an approximation of true birth probabilities, B_*i*, corrected_), we fit three additional Cormack–Jolly–Seber models to the data from Hastings (2017) to reevaluate the best model for describing the effect of maternal age on early pup survival. Hastings (2017) used daily resighting data throughout the pupping season to provide estimates of early pup survival at Forrester by year (2007–2014), pup age (weeks 0–1, weeks 2+), and maternal age (two age categories: mothers aged 5–7 vs. 8+), but only fit models with maternal age as a discrete variable. We refit models to determine if a continuous maternal age variable (linear, quadratic, or basis spline) provided a better fit. The resulting best estimates of early pup survival at Forrester ($\varphi_{\mathrm{pup}\;3\;\text{weeks}}=\varphi_{\mathrm{pup}\;1–2\;\text{weeks}}^{2}*\varphi_{\mathrm{pup}\;3\mathrm{rd}\;\text{week}}$) for an average year (2007, Hastings, 2017) were used to correct estimates of B_*i*_ using: B_*i*, corrected_ = B_*i*_/*φ*_pup 3 weeks, maternal age *i*_.

We included the derived age‐specific estimates of pup production (B_*i*_, the proportion of females alive that were with pups at the end of the pupping season) and survival in simple, deterministic Leslie matrix models using the R package *popbio* (Stubben & Milligan, 2007) to determine the effects of reproductive patterns on population trend estimates separately for the south region (Forrester and Hazy pooled), White Sisters and Graves Rocks. Population growth rate was estimated as the dominant eigenvalue of the Leslie matrix comprised of fully age‐specific fecundity and survival schedules to age 30, assuming constant values after age 25, for an average year before the PMH and after the PMH. We calculated the 95% CI of estimated population trend using R and the delta method following Skalski et al. (2007) and Bowles et al. (2015). It was appropriate to use B_*i*_, the proportion of females alive that were with pups at the end of the pupping season which included early pup mortality, in these models because pups were marked at ~3 weeks of age and therefore first‐year survival excluded early pup mortality. Therefore, these parameters provided complimentary survival and reproductive information for models.

## 3 RESULTS

**FIGURE 2:**
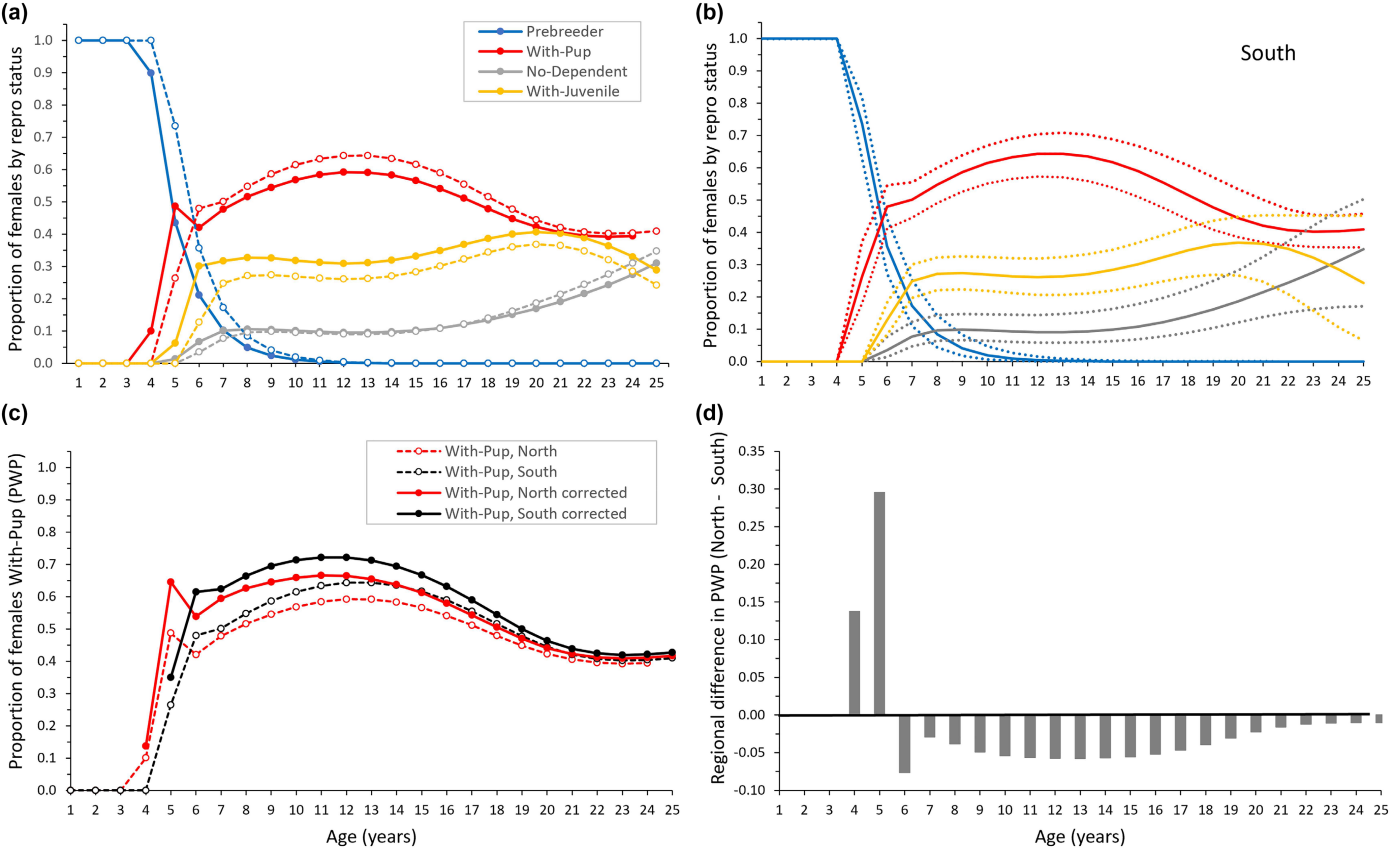
Proportion of Steller sea lion females in Southeast Alaska (2005–2019) by reproductive state at the end of the pupping season by age and natal region. (a) Natal regions were South (Forrester and Hazy in open circles/dashed line) and North (White Sisters and Graves Rocks in solid circles and solid lines; Figure 1). (b) Estimates for natal region South with 95% CI. (c) Estimates of true birth probabilities: proportion of females With‐Pup was corrected for early pup mortality from birth to age 3 weeks (see text). (d) Regional difference in the proportion of females with pup (North–South). PWP, Proportion With‐Pup. Estimates shown are for years <2011.

Our most important results (detailed below) included: important age variation in reproductive output was observed as a gradual increase in proportion of females with pup from the age of first recruitment to ~12 years of age, followed by gradual senescence to at least age 20 (Figure 2). In a typical year before the PMH at peak reproductive ages, 0.60/0.65 of females (north/south) were with pup at the end of the pupping season, 0.30/0.25 were with juvenile, and 0.10 (both regions) were without a dependent. Important regional variation included earlier recruitment (age 4 rather than age 5) but thereafter slightly lower pup production and higher juvenile retention in the productive north region compared to the south (Figure 2). Important year variation included consistently lower pup production in both regions after the PMH (>2014; Figure 5), which if sustained in the south would result in population decline. We also found no evidence that reproductive state at the end of the pupping season affected female survival pre‐ or post‐PMH.

### 3.1 Reproductive performance and female survival

3.1.1

**FIGURE 3:**
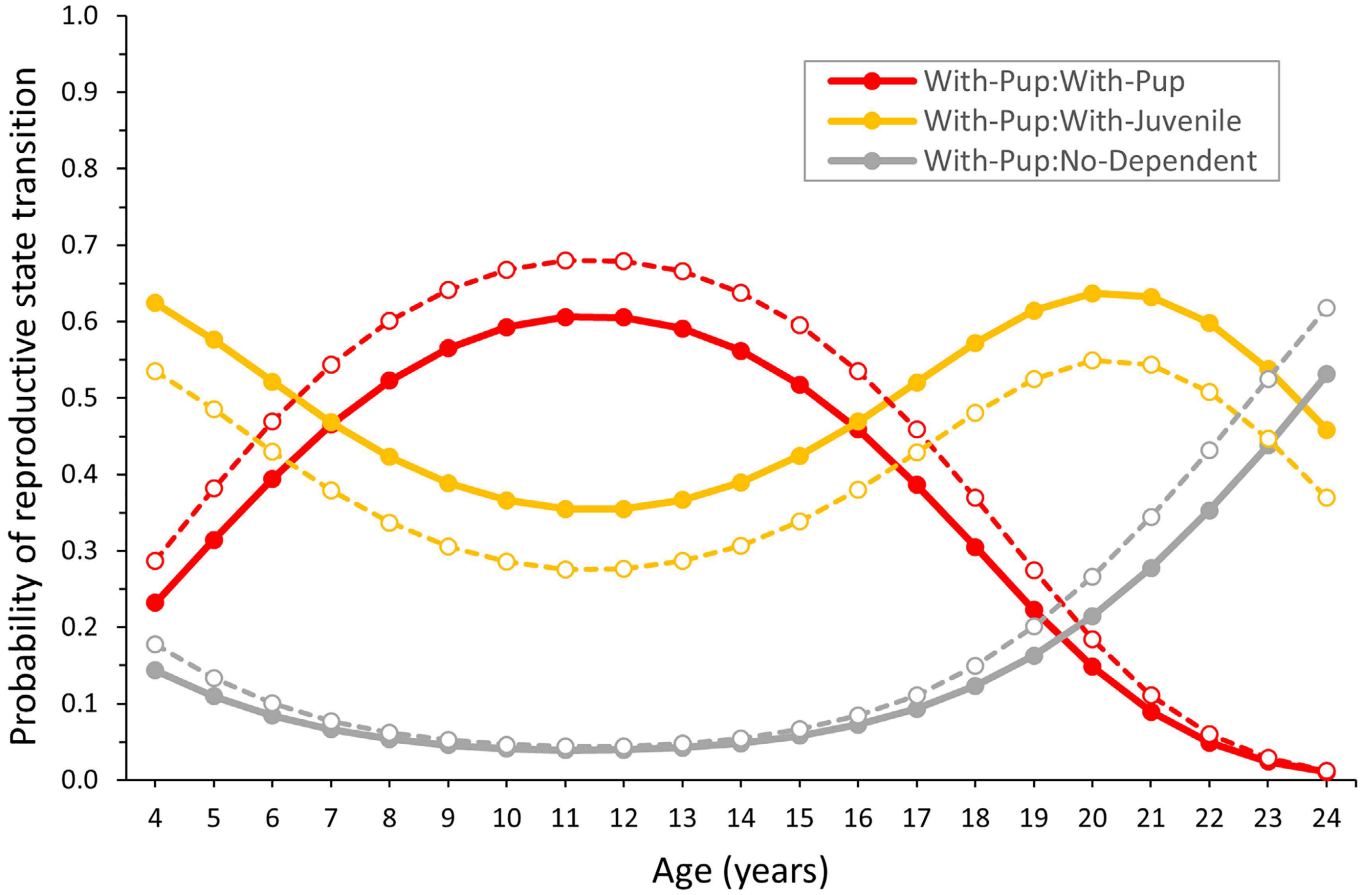
Probability of transitioning reproductive states for Steller sea lion females With‐Pup in Southeast Alaska by age and natal region. Natal regions were South (Forrester and Hazy in open circles/dashed line) and North (White Sisters and Graves Rocks in solid circles and solid lines; Figure 1). Estimates plotted are for the years <2011.

Concerning age and region effects on reproduction, females began pupping at younger ages in the north (age 4) than in the south (age 5; Figure 2a), which resulted in 0.14 and 0.30 more females With‐Pup in the north than in the south at ages 4 and 5, respectively (Figure 2d). After the first age of recruitment for each region, the subsequent rate of recruitment was high and the same for both regions (0.515; Table 2). By age 8/9 (north/south), ~95% of females had recruited (Figure 2). In addition to regional differences in recruitment age, With‐Pup females in the north were also more likely to have a dependent juvenile the year after producing a pup than their counterparts in the south (+0.09, With‐Pup:With‐Juvenile; Figure 3), which were more likely to have a pup in the year after producing a pup (With‐Pup:With‐Pup; Figure 3).

**TABLE 2:** Estimates of female resighting probabilities per day at rookeries (estimates for 2008 are shown) and the probabilities of transitioning between reproductive states and moving between rookeries (R) and haulouts (H) for female Steller sea lions in Southeast Alaska, 2005–2019.

	Estimate (95% CI)
Female resighting probability, at rookeries	
With‐Pup, Forrester	0.456 (0.410–0.502)
With‐Pup, Hazy	0.439 (0.383–0.498)
With‐Pup, White Sisters	0.541 (0.478–0.600)
With‐Pup, Graves Rocks	0.651 (0.574–0.720)
With‐Juvenile, South, first sighting	0.058 (0.045–0.075)
With‐Juvenile, South, subsequent sighting	0.407 (0.330–0.489)
With‐Juvenile, North, first sighting	0.084 (0.062–0.113)
With‐Juvenile, North, subsequent sighting	0.506 (0.410–0.603)
No‐Dependent, first sighting	0.579 (0.415–0.728)
No‐Dependent, subsequent sighting	0.377 (0.290–0.470)
Prebreeder or juvenile, South, first sighting	0.249 (0.205–0.300)
Prebreeder or juvenile, South, subsequent sighting	0.375 (0.315–0.438)
Prebreeder or juvenile, North, first sighting	0.374 (0.319–0.431)
Prebreeder or juvenile, North, subsequent sighting	0.519 (0.453–0.582)
Reproductive state transition probability	
Prebreeder^b^:With‐Pup (1994–1995 cohorts)	0.816 (0.298–0.979)
Prebreeder:With‐Pup, North, age 3	0.100 (0.047–0.200)
Prebreeder:With‐Pup, North, age 4+^a^	0.515 (0.425–0.603)
Prebreeder:With‐Pup, South, age 3	0.000
Prebreeder:With‐Pup, South, age 4	0.264 (0.179–0.371)
Prebreeder:With‐Pup, South, age 5+^a^	0.515 (0.425–0.603)
With‐Juvenile:With‐Pup	0.483 (0.367–0.595)
With‐Juvenile:With‐Juvenile	0.328 (0.223–0.444)
With‐Juvenile:No‐Dependent	0.188 (0.107–0.305)
No‐Dependent:With‐Pup	0.857 (0.643–0.952)
No‐Dependent:No‐Dependent	0.143 (0.048–0.357)
Movement transition probability	
H:R, juvenile female	0.322 (0.276–0.373)
H:R, Prebreeder (>age 3)	0.678 (0.596–0.751)
H:R, No‐Dependent	0.793 (0.577–0.915)
H:R, With‐Pup	0.829 (0.770–0.875)
H:R, With‐Juvenile	0.727 (0.472–0.888)
R:H, juvenile female	0.331 (0.245–0.429)
R:H, Prebreeder (>age 3)	0.160 (0.118–0.213)
R:H, No‐Dependent	0.020 (0.003–0.110)
R:H, With‐Pup	0.009 (0.003–0.028)
R:H, With‐Juvenile	0.554 (0.338–0.751)

*Note*: North/South = natal regions North (White Sisters or Graves Rocks) or South (Forrester or Hazy Islands; Figure 1). Reproductive state transitions between years were Prebreeder:With‐Pup (recruitment), No‐Dependent:With‐Pup, With‐Juvenile:With‐Pup, With‐Juvenile:No‐Dependent (see Appendix 3; for age‐dependent reproductive state transitions—With‐Pup:With‐Juvenile, With‐Pup:No‐Dependent—see Figure 3). Movement transitions were from haulout to rookery (H:R) between years by age/reproductive state in the next year, or from R:H within a year by age/reproductive state in the current year. Age/reproductive states for movement parameters were Prebreeder 4+ (nulliparous age 4+), With‐Pup, With‐Juvenile, No‐Dependent (parous), or juvenile female (age 0–3). ^a^Same estimate. Recruitment of cohorts 1994–1995^b^ was fit separately and was not true recruitment probability but was the probability of recruiting into the population of knowable state per year after 2004. For annual variation in resighting probabilities see Figure 6.

In addition to recruitment probability, age effects were important predictors of transitions from With‐Pup. With‐Pup:With‐Pup was highest at middle ages, and With‐Pup:With‐Juvenile and With‐Pup:No‐Dependent were the highest for the oldest females (especially in With‐Pup:No‐Dependent after age 18–20) and the youngest females (Figure 3). Transitions from With‐Juvenile and No‐Dependent did not vary with age or region (Table 2, Appendix 4). Although model selection suggested some age variation in the transition probability No‐Dependent:With‐Pup, estimates of 0 and 1 (i.e., at the parameter‐space boundary and possibly unreliable) were produced (perhaps due to small numbers of No‐Dependent females; Figure 2a). Therefore, we modeled this parameter as constant with age. Probabilities of transitioning to No‐Dependent were low (No‐Dependent:No‐Dependent = 0.143; With‐Juvenile:No‐Dependent = 0.188, and With‐Pup:No‐Dependent ~0.05 until age 18–20; Figure 3, Table 2). With‐juvenile females more often produced a pup the next year (0.483) than retained their juvenile for another year (0.328, Table 2).

The proportions of females with pup at the end of the pupping season (B_*i*_) were higher in the north than in the south at age 4–5 due to earlier recruitment, but from age 6 until ages 18–20, B_*i*_ in the north was ~0.05 lower than in the south (Figure 2a). Because of recruitment and higher transition probabilities With‐Pup:With‐Juvenile and With‐Pup:No‐Dependent for younger mothers (Figure 3), females did not reach peak pup production age until 12–13 (Figure 2). In a typical year pre‐PMH at the end of the pupping season (including early pup mortality), ~0.60 to 0.65 of prime‐aged females were with pup, ~0.25 to 0.30 were with juvenile, and ~0.10 had no dependent (Figure 2a, Table S1). Therefore, most parous females were with a dependent from year to year, until age 18–20 when transitioning to No‐Dependent became more common (Figures 2 and 3). Senescence in birth probabilities was evident after age 12–13: by age 25, B_*i*_ was ~0.40, J_*i*_ was ~0.30, and N_*i*_ was ~0.30, in both regions (Figure 2a).

**FIGURE 4:**
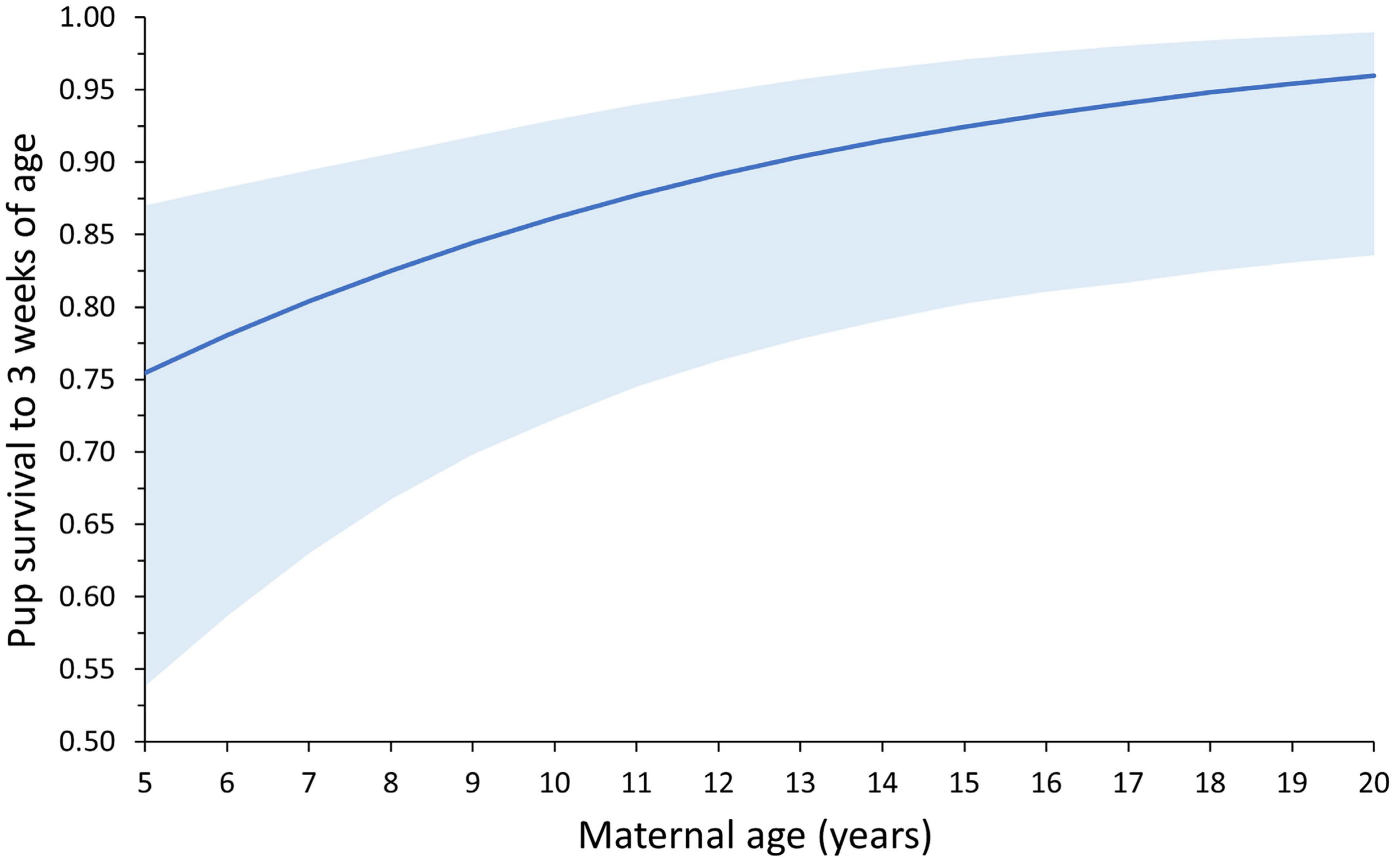
Survival of Steller sea lion pups to 3 weeks of age based on maternal age at Forrester Islands, Southeast Alaska, from 2007 to 2014. Models were refit to the data from Hastings (2017) to also include models that estimated effect of maternal age on pup survival as a continuous variable (see text). Estimates for an average/good year (2007) are shown. Blue ribbon is 95% CI.

By refitting data from Hastings (2017) to provide a simple correction to B_*i*_ for early pup mortality, the model with a linear trend in pup survival with maternal age (ages 5–20) had the most support (AIC Weight = 0.46, vs. 0.20 and 0.17 for quadratic and spline fits, and 0.12 and 0.06 for two and three age categories, respectively). Early pup survival ranged from 0.76 for pups of age 5 mothers to 0.96 to pups of age 20 mothers (Figure 4). This correction was applied to estimates for both the north and south regions and produced estimates of true birth probabilities B_*i*, corrected_ of 0.722 in the south and 0.664 in the north, at age 12, and shifted the peak reproductive ages slightly earlier (ages 10–12; Figure 2c).

3.1.2

**FIGURE 5:**
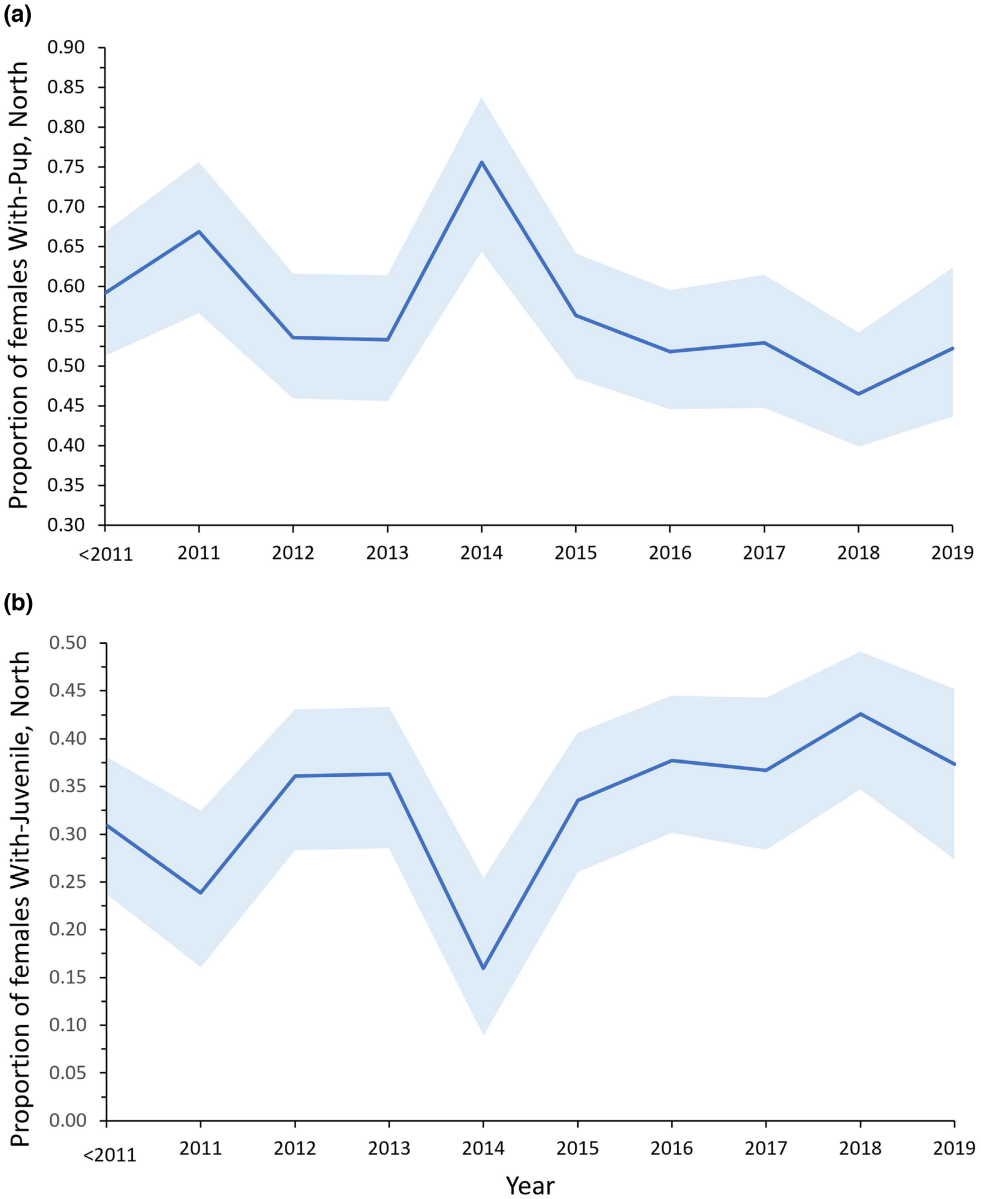
Annual variation in the proportion of female Steller sea lions (a) With‐Pup or (b) With‐Juvenile in Southeast Alaska, 2005–2019. Blue ribbons are 95% CI. Estimates plotted are for females aged 12 (peak pupping age, see Figure 2a) born in the North (White Sisters or Graves Rocks; Figure 1).

Concerning year effects on reproduction, cohort variation in recruitment rates (five cohorts) was not supported, but year variation in the transition probability With‐Pup:With‐Juvenile was important (Figure 5, model 72, Appendix 4). Pup production (B_*i*_) was high in 2014, and consistently lower after initial ocean warming in summer 2014 (Figure 5, Table S2). Compared to estimates <2014 (from a post hoc model with subsets of years pre‐ and post‐PMH pooled, $\bar{x}$_north_ = 0.567, 95% CI: 0.496–0.631, females aged 12), the average B_*i*_ among years was ~0.061 lower >2015 ($\bar{x}$_north_ = 0.506, 0.444–0.560), resulting in a greater proportion of prime‐aged females with juveniles >2015, whereas proportion with no dependent did not change appreciably (Figure 5, Table S2).

3.1.3

Concerning female survival, age, region, and year effects on female survival are well documented (Hastings et al., 2023), and survival estimates in this study were consistent with past analyses of these data (Hastings et al., 2011, 2018, 2023, estimates provided in Table S3). In this paper we addressed female survival in relation to reproductive state at the end of the pupping season. No models that included the effect of reproductive state on female survival overall, by age‐class, during poor survival years or after ocean warming in 2014 were supported (Appendix 4).

Concerning potential effects of reduced reproduction on population trend, when the resulting age‐specific reproductive and survival probabilities were included in a Leslie matrix model using average pre‐PMH values (pooled years <2011, Tables S1 and S3), estimated population growth rates were: $\hat{r}$‐south = −0.007 (95% CI: −0.017, 0.002), $\hat{r}$‐White Sisters = 0.025 (0.014, 0.035), and $\hat{r}$‐Graves Rocks = 0.047 (0.031, 0.062). Reduced reproduction following the PMH was large enough to affect the estimates of population trend. We replaced the B_*i*_ values in the Leslie matrix with those for 2016 (because they were similar to average values >2014; see Figure 5). If this lower average reproductive output is sustained, an $\hat{r}$‐south of −0.015 (−0.025, −0.006) would result, indicating a population decline in the south.

### 3.2 Nuisance parameters: Female resighting probabilities, offspring detection probabilities, and female movement probabilities

3.2.1

**FIGURE 6:**
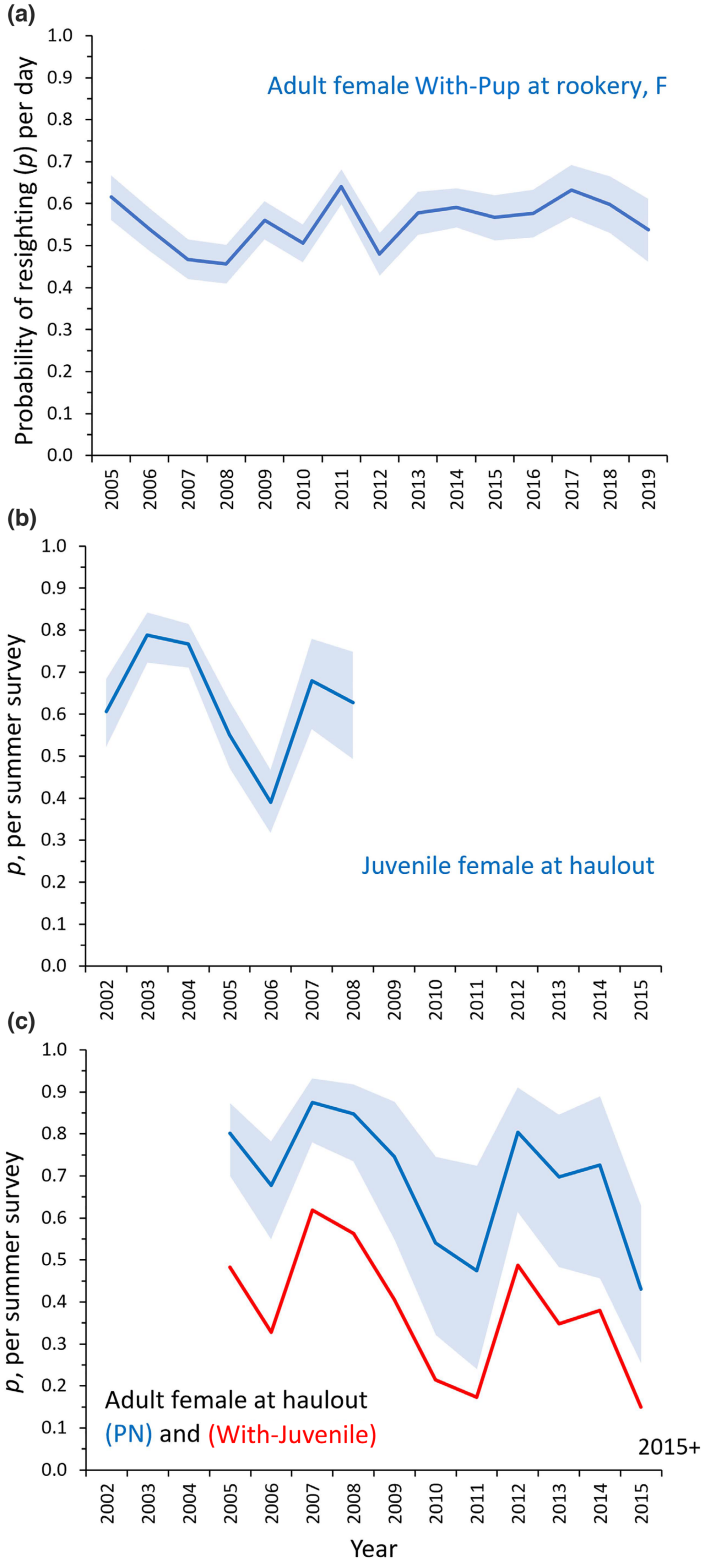
Annual variation in probability of resighting per day at rookery (a) or per summer survey at haulout (b, c) for Steller sea lion females in Southeast Alaska, 2002–2019. Blue ribbons are 95% CI. Estimates shown are for (a) females With‐Pup at rookery (natal rookery Forrester Island F), (b) juvenile females (age 1–3), and (c) adult female Prebreeder 4+ (age 4+) or No‐Dependent (parous) pooled (in blue, PN) or With‐Juvenile (in red). In (c), resighting rate was pooled for years 2015+ due to low number of resights per year.

For resighting probabilities of females at rookeries, probabilities were the lowest ($\bar{x}$ = 0.33–0.47) and similar for Adult Prebreeders 4+ and juvenile females aged 0–3 (Table 2, Appendix 4). Resighting probabilities of females With‐Pup were also highest for smaller northern rookeries (e.g., +0.21 and + 0.10 for Graves Rocks‐born and White Sisters‐born, respectively, compared to southern‐born females With‐Pup, Table 2). At haulouts, annual variation in female resighting probabilities was high, where effort varied annually, compared to less annual variation in resighting probabilities at rookeries, where effort was also significant in the model but more consistent annually compared to haulouts (Figure 6).

3.2.2

At rookeries, resighting probabilities for With‐Pup females were high ($\bar{x}$ = 0.54–0.73 per day among rookeries) and did not vary for first versus subsequent resightings. Also at rookeries, the resighting probabilities for With‐Juvenile females were much reduced for first ($\bar{x}$ = 0.07 per day) versus subsequent sightings ($\bar{x}$ = 0.46 per day) and this predictor had the greatest effect on ΔAIC for this parameter (Table 2, Appendix 4). Resighting probability was high for first sightings of No‐Dependent females ($\bar{x}$ = 0.67, all rookeries were similar), but, unlike With‐Juvenile females, was slightly lower (−0.19) for subsequent sightings. Juvenile females aged 0–3 and Adult Prebreeders 4+ had lower resighting probabilities at rookeries ($\bar{x}$ = 0.33–0.47 among rookeries) but, similar to With‐Juvenile females, resighting probability was higher on subsequent than first sightings (+0.14, Table 2). At haulouts, resighting probabilities were 0.24–0.30 higher for No‐Dependent and Prebreeder 4+ adult females compared to With‐Juvenile females and juvenile females aged 0–3 (Figure 6b,c).

For offspring detection proabilities, only regional and reproductive state effects were fit: probabilities were higher for With‐Juvenile (0.56, 95% CI: 0.49–0.61, all rookeries pooled) than for With‐Pup females. Pup detection probabilities varied among natal rookeries and were the lowest for females born at the large southern rookeries (Forrester = 0.34, 0.31–0.37, Hazy = 0.39, 0.35–0.44, White Sisters and Graves Rocks pooled: 0.48, 0.44–0.53). For movement parameters, the global model could not be simplified (Appendix 4). Resulting movement estimates were reasonable: juvenile females 0–3 used rookeries less than other groups, and juvenile females aged 0–3 and With‐Juvenile adult females moved between rookeries and haulouts more than other groups in the same survey year (Table 2).

## 4 DISCUSSION

We estimated true birth probabilities at peak reproductive age 12 were 0.722 and 0.664 for females born in southern and northern Southeast Alaska, respectively. True birth probabilities may be higher than 0.664 at smaller and less dense northern rookeries, if neonatal survival is higher than at the large, dense Forrester Island rookery, which had particularly high early pup mortality (Hastings, 2017; Kaplan et al., 2008; Maniscalco et al., 2008) and was the source of our neonatal mortality correction estimate. These estimates are similar to the 0.70 estimate for branded Forrester females in 2004 at ages 9–10 (Taylor & Boor, 2012) and for females of all ages at Chiswell Island, Alaska from 2003 to 2012 (Maniscalco et al., 2014). If a similar pattern occurs with age for Chiswell Island females, birth probabilities at peak ages would be higher at that rookery than in Southeast Alaska. A recent estimate for Steller sea lion females in the northern Gulf of Alaska also suggests higher peak reproductive output in this area (0.80, Warlick et al., 2022). Birth probability estimates for females in the Gulf of Alaska based on cross‐sectional data were 0.63 during 1975‐1978 (pre‐decline) and were 0.55 in 1985 and 1986 during a period of dramatic decline, due to high rates of late‐term abortions for younger, lactating females (Pitcher & Calkins, 1981; Pitcher et al., 1998). Our estimates cannot be directly compared to these historical values without assuming similar population age structures, but the average birth probability for ages 6–15 in our study (0.630 and 0.683 for the north and south, respectively) was similar to pre‐decline values.

Our estimates of peak reproductive output in Steller sea lions in Southeast Alaska were moderately high compared to those for other otariids. Our estimates were similar to published estimates for subantarctic fur seals, *Arctocephalus tropicalis*, on Amsterdam Island (0.721 at age 12; Dabin et al., 2004) and New Zealand fur seals, *Arctocephalus forsteri*, at Kangaroo Island (0.60–0.70 at age 8+; McKenzie et al., 2007), and much higher than the endangered Galapagos sea lion, *Zalophus wollebaeki* (0.40–0.48 at ages 6+; Kalberer et al., 2018). In contrast, our estimates at peak ages were lower than published estimates for the California sea lion, *Zalophus californianus* (0.77–0.80; Hernández‐Camacho et al., 2008; Melin et al., 2012), the endangered New Zealand Sea Lion, *Phocarctos hookeri* (~0.75 to 0.88 at age 7+; Childerhouse et al., 2010) and the Antarctic fur seal, *Arctocephalus gazella* (0.80 at ages 7–9 at Bird Island; Lunn et al., 1994; 0.90 at ages 8–16 at Livingston Island; Schwarz et al., 2013).

Although the combined survival probabilities and reproductive output was sufficient for population stability or growth in Southeast Alaska pre‐PMH (Mathews et al., 2011; Pitcher et al., 2007), we found age‐related demographic processes, particularly senescence, may be an important component of female reproductive strategies. Reproductive senescence is expected for female mammals (Comizzoli & Ottinger, 2021) and a sharp drop in birth or pregnancy rates at older ages was observed in other otariid species (starting at ages 13–17; Dabin et al., 2004; Eberhardt, 1985; Hernández‐Camacho et al., 2008; Melin et al., 2012). We observed a gradual drop in birth probabilities after the peak age (~−0.20 reduction from ages 12 to 21), most similar to that observed for Antarctic fur seals (Lunn et al., 1994), although the rate of decline in Steller sea lions after age 20 requires more study (*n* < 20 females seen per age after age 20; Appendix 2).

Reduced birth probabilities in older females were associated with increased retention of juveniles and also steep increases in the probability of being without any dependent (Figure 3), suggesting failure in reproductive physiology with age. Usually ~0.10 of females were without a dependent in a year; this proportion increased steeply after age 17–18 (to perhaps 0.30; Figure 2a). The physiological mechanisms responsible for female reproductive aging (i.e., infertility) are very similar across vertebrate species, at the level of the whole organism, reproductive organs and germ cells, including: the depletion of egg reserves, loss of ovarian function, changes to the uterine environment, loss of cycles of reproductive hormones (especially circulating estradiol), decreased steroid production, and a decline in the estrogen‐dependent endocrine and behavioral responses that drive reproduction (Ottinger, 2010).

Causal mechanisms underlying reproductive senescence in Steller sea lions require study, but neonatal survival remained high for the oldest mothers (Figure 4), suggesting adequate maternal condition during the neonatal period for older females that produced pups (and/or the important role of maternal experience in early pup survival at the rookery). In an historical sample collected in 1975–1978 and 1985–1986, pregnancy rates were low for females aged 15+ compared to prime‐aged females and all three of females aged 21–30 experienced reproductive failures for undetermined reasons (table 2 in Pitcher & Calkins, 1981; table 6 in Calkins & Goodwin, 1988). However, sample size of oldest females was very small (*n* = 13), which also may have precluded the ability to determine whether body condition declined for the oldest females in that sample (Pitcher et al., 1998). However, parturition dates were later for the oldest mothers (parturition dates became earlier from ages 5 to 12 and then became later from ages 12 to 20; Hastings & Jemison, 2016; but see Maniscalco & Parker, 2018) and later parturition dates were associated with poor body condition in other species (reviewed by Hastings & Jemison, 2016).

As commonly seen in otariids (Lunn et al., 1994; McKenzie et al., 2007; Melin et al., 2012), the recruitment rate (based on reproductive state at the end of pupping season) was high (0.515 per age) and recruitment occurred mainly over a few ages (ages 5–7; Figure 2a). This figure is likely an underestimate of recruitment rate based on all live births, because our recruitment rate estimates were based on pup production at the end of the pupping season and early pup mortality is higher in younger than older mothers (Figure 4). Compared to prime‐aged females, reduced pup production by young females resulted from recruitment and high neonatal mortality (Figure 4) and was associated with greater retention of their juveniles (Figure 3). High neonatal mortality of pups born to young mothers delayed the peak output of “viable” pups (pups that survived the period of high neonatal mortality at <3 weeks of age; Hastings, 2017) from ages 8–15 to ages 10–15 (Figure 2c). The probability of young mothers retaining a juvenile was up to 0.25 higher, but the probability of skipping pupping without a dependent juvenile was not appreciably higher than probabilities for prime‐aged females (Figure 3). We suspect higher probability of retaining the juvenile results from higher abortion rates in younger lactating females, which are particularly affected by nutritional stress (Pitcher et al., 1998). More study is required to determine if this pattern is also associated with delayed weaning for offspring of young mothers, perhaps due to the smaller size and slower growth of their pups, which would allow them to reach a weaning size threshold important for future survival and reproduction, a key driver in population dynamics, the behavior of mothers and offspring, and reproductive strategies in Steller sea lions (Hastings et al., 2021). Reduced reproductive output of young female Steller sea lions is expected as asymptotic body mass is reached at later ages than recruitment (~age 13 in Steller sea lions: Winship et al., 2001), a common pattern in female pinnipeds (Boltnev & York, 2001; Dabin et al., 2004; Grandi et al., 2010; Laws, 1956), and probability of pregnancy during late gestation is strongly dependent on female mass and condition (Pitcher et al., 1998).

Regional variation in population dynamics suggests a favorable environment in northern Southeast Alaska, and larger body size of northern‐born pups and smaller home ranges suggests animal density may be low relative to environmental productivity in the north (Hastings et al., 2011; Jemison et al., 2018; Mathews et al., 2011). Formal studies of regional variation in sea lion prey abundance and composition in Southeast Alaska are lacking but high productivity in the north is suspected due to rapid and recent deglaciation in Glacier Bay resulting in new habitat (Mathews et al., 2011). This area is characterized by high levels of mixing, primary and secondary productivity, and dense forage fish schools which also concentrate in shallower depths during the day perhaps providing more efficient foraging for sea lions (reviewed by Rehberg et al., 2018). Areas of strong tidal currents also concentrate prey and serve as important corridors for migrating Pacific salmon; protections afforded by Glacier Bay National Park (including the Graves Rocks rookery) may also minimize threats and harassment to sea lions (reviewed by Rehberg et al., 2018).

A nutritional component for regional differences may be further supported by earlier recruitment of females at ages 4–5 in the north than in the south (Figure 2). Earlier recruitment in long‐lived mammals generally improves fitness, promotes population growth, and is indicative of high food abundance relative to animal density (Cole, 1954; Fowler, 1987; Stearns, 1976). However, after age 5, pup production averaged ~0.05 lower in the north than in the south, associated with a slightly greater retention of juveniles (Figure 3) likely due to higher offspring survival in the north than in the south (+0.11 and +0.07 from age 0–1 and 1–2, respectively, for northern‐born offspring, Hastings et al., 2011). In fact, juvenile survival to age 4 was higher in northern Southeast Alaska than in all other areas studied from Oregon through Russia (Wright et al., 2017). Therefore, high population growth in the north (Mathews et al., 2011) may be driven by not only immigration (Jemison et al., 2013, 2018) and high juvenile survival (Hastings et al., 2011) but also younger ages of first reproduction rather than higher annual reproductive output. In addition, weaning ages were similar between regions within Southeast Alaska but sea lions in Southeast Alaska were smaller and weaned later than their counterparts west of Cook Inlet in the northern Gulf of Alaska, perhaps due to a less productive and/or more variable environment in which females may exist closer to the edge of their physiological capacity for producing successful offspring (Hastings et al., 2021). This idea is supported by our observation that females produce offspring earlier but do not produce more offspring even in productive areas of Southeast Alaska, perhaps due to body‐size and growth constraints and the need to commonly invest >1 year in offspring to ensure they are able to reach an appropriate size for successful weaning (Hastings et al., 2021, this study).

Surprisingly, a cost of reproduction on female survival was not detected in our study and causes for low adult female survival in 2014–2016 (Hastings et al., 2023) may have similarly impacted females with and without dependents. If negative changes to the prey field during the PMH (Suryan et al., 2021) contributed to adult female mortality, we expected that females with dependents would be especially impacted. If our result is correct, it suggests female Steller sea lions are physiologically fine‐tuned to their environment: during a period of steep population decline from 1975 to 1986, lactating females aborted their fetuses during mid‐to‐late gestation (essentially all mature females were pregnant and implanted annually by late fall), reducing birth rates by 0.08 and this was accompanied by smaller body size of females (Calkins et al., 1998; Pitcher & Calkins, 1981; Pitcher et al., 1998). Also, during that decline, juvenile mortality was high, with potential periods of high adult mortality (Pendleton et al., 2006; York, 1994; York et al., 1996). We saw no evidence of a failure to properly buffer adult survival and offspring support or production during the PMH, similar to a study at Chiswell Island before the PMH (Maniscalco et al., 2014). However, reproductive status at the end of the pupping season may not sufficiently reflect survival costs and energy burdens over the next year: the energy balance of a female that loses her dependent shortly after the pupping season (due to death or weaning) may be similar to that of a female without a dependent at the end of the pupping season. Costs of reproduction may also be masked by effects of individual quality (Chambert et al., 2013; Hamel et al., 2009), suggesting that more study is needed to tease apart these potential confounding factors.

Although we found no evidence that low adult female survival in 2014–2016 was related to reproductive state at the end of the pupping season, pup production remained consistently at lower levels (−0.06 from the mean from 2005 to 2013) in Southeast Alaska following ocean warming in 2014 (from 2015 to 2019; Figure 5). Warm surface water reached the coast of Southeast Alaska in spring–summer of 2014 with peak temperatures in 2015–2016, cooled in 2017 and warmed again in spring 2019 (Bond et al., 2015; Chen et al., 2021; Danielson et al., 2022). Viable pup production was annually variable (up to ~0.20) and was particularly high in 2014 for unknown reasons (Figure 5). After 2014, the lower numbers of pups produced was associated with a greater probability of females retaining their juveniles but not an appreciably greater probability of females being without any dependent (Figure 5, Table S2). Together with historical studies, these patterns suggest that reduced reproductive output, and possibly greater retention of juveniles, during periods of poor prey conditions is an important strategy in Steller sea lions in Southeast Alaska. This may be due to selection for fine‐tuning of reproductive output based on nutritional status to improve the probability of producing pups under good conditions throughout their lifetimes in a variable and less productive environment. However, the reduction in reproduction we documented, if sustained with survival probabilities remaining at current levels, could reduce population growth and the population in southern Southeast Alaska may no longer be stable but declining at a rate of −0.015 (−0.025, −0.006) per year.

## AUTHOR CONTRIBUTIONS

**Kelly K. Hastings:** Conceptualization (equal); data curation (lead); formal analysis (lead); investigation (equal); methodology (equal); writing – original draft (lead); writing – review and editing (equal). **Lauri A. Jemison:** Conceptualization (equal); investigation (equal); methodology (equal); project administration (lead); writing – review and editing (equal). **Grey W. Pendleton:** Conceptualization (equal); investigation (equal); methodology (equal); writing – review and editing (equal). **Devin S. Johnson:** Conceptualization (equal); investigation (equal); methodology (equal); writing – review and editing (equal). **Thomas S. Gelatt:** Conceptualization (equal); investigation (equal); methodology (equal); writing – review and editing (equal).

## CONFLICT OF INTEREST STATEMENT

None declared.

## Supporting information


Table S1.
Table S2.
Table S3.

## Data Availability

Data are available in the Dryad Digital Repository: https://doi.org/10.5061/dryad.4qrfj6qgc.

## APPENDIX 1 Example capture histories for Steller sea lion females in Southeast Alaska. Codes for reproductive state: B = With‐Pup, J = With‐Juvenile, u = uncertain, P = Prebreeder (only possible on the first release). Codes for location: R = rookery, H = haulout, where only H was possible from 2001 to 2004 and on occasion 4 per year after 2004; R was possible only for occasion 1–3 per year after 2004. 0 = female not seen. After 2004, survival was estimated and reproductive state transitions were possible only between occasion 4 in year *x* and occasion 1 in year *x* + 1. Reproductive surveys (conducted in late June‐mid July) began in 2005 with three daily surveys per annual summer survey at the rookery (in yellow boxes) and one occasion at the haulout (summarizing any resighting at a haulout during the survey window that year, in gray boxes). A time‐varying covariate, “seen before” (sb), was included for rookery occasions to allow female resighting probability to vary between the first and subsequent resightings within a year (see text, instances when females were seen on the previous rookery occasion are colored red).

**Table:**

Female	2001	2002	2003	2004	2005				2006				2007				2008				2009				2010				…	2019			
					1	2	3	4	1	2	3	4	1	2	3	4	1	2	3	4	1	2	3	4	1	2	3	4		1	2	3	4
F1114	HP	Hu	Hu	0	0	Ru	0	0	0	Ru	0	Hu	0	0	0	0	RB	0	0	0	RJ	RJ	Ru	0	0	0	0	0	…	0	0	0	0
F1116	HP	0	Hu	Hu	Ru	Ru	0	0	Ru	Ru	0	0	Ru	Ru	0	0	RB	Ru	Ru	0	0	0	0	Hu	0	0	0	0	…	RB	Ru	0	0
*Time‐varying covariate “seen before” (sb)*																																	
F1114:sb	0	0	0	0	0	0	1	0	0	0	1	0	0	0	0	0	0	1	1	0	0	1	1	0	0	0	0	0	…	0	0	0	0
F1116:sb	0	0	0	0	0	1	1	0	0	1	1	0	0	1	1	0	0	1	1	0	0	0	0	0	0	0	0	0	…	0	1	1	0

## APPENDIX 2 Numbers of marked Steller sea lion females resighted per age at rookeries and haulouts in Southeast Alaska from 2005 to 2019 by the most definitive reproductive status observed per location per year. Reproductive states were With‐Pup, With‐Juvenile, and Uncertain.

**Table:**

Age	Rookery				Haulout		
	With‐pup	With‐Juv	Uncertain	All	Uncertain	With‐Juv	All
1			63	63	76		76
2			53	53	97		97
3			84	84	91		91
4	8	1	168	177	95		95
5	58		149	207	38		38
6	73	9	124	206	23	3	26
7	62	8	101	171	22	6	28
8	79	16	83	178	19	12	31
9	90	7	95	192	11	6	17
10	99	11	93	203	9	9	18
11	80	17	123	220	15	7	22
12	83	10	105	198	14	7	21
13	77	16	86	179	7	8	15
14	67	20	70	157	6	3	9
15	47	8	51	106	5	2	7
16	25	10	50	85	1		1
17	26	5	36	67	2	2	4
18	15	1	24	40	1	1	2
19	5	2	11	18	3		3
20	6	1	13	20	1		1
21	1	1	10	12	1		1
22	1	1	12	14	2		2
23		1	6	7			
24			6	6			
25			1	1			

## APPENDIX 3 Estimation of six possible reproductive state transitions used to examine age‐specific reproductive performance of female Steller sea lions in Southeast Alaska, 2005–2019. The probability of remaining in the same state was estimated as the difference of the other row‐wise probabilities which, as multinomial variables, must sum to 1 (in red in yellow boxes). Impossible reproductive state transitions (in gray boxes) were fixed to 0. Four possible reproductive states were: P = Prebreeder (nulliparous), With‐Pup, With‐Juvenile, and No‐Dependent (parous).

**Table:**

From state	To state			
	Prebreeder	With‐Pup	With‐Juvenile	No‐Dependent
Prebreeder	1	e^*β*1^	0	0
With‐Pup	0	1	e^*β*2^	e^*β*3^
With‐Juvenile	0	e^*β*4^	1	e^*β*5^
No‐Dependent	0	e^*β*6^	0	1

## APPENDIX 4 Model selection results for reproductive performance of female Steller sea lions in Southeast Alaska, 2005–2019. np = number of parameters, nr = natal rookery (see four rookeries in Southeast Alaska; Figure 1); Reg = natal region (North: White Sisters or Graves Rocks or South: Forrester or Hazy Islands; see Figure 1). H = Haulout, R = rookery, yr = year, grp = demographic group by location: adult females at rookeries = Prebreeder4+ nulliparous (ARP), With‐Pup (ARB), With‐Juvenile (ARJ), or No‐Dependent parous (ARN). Juvenile female at rookery = VR (aged 0–3); adult female at haulout = With‐Dependent (AHBJ) or No‐Dependent parous or Prebreeder4+ (AHNP). ARPV pooled groups ARP and VR. sb = seen before that year. nr2 = three categories for natal rookery: Forrester, Hazy, and White Sisters = Graves Rocks (see Figure 1). B = With‐Pup, J = With‐Juvenile, N = No‐Dependent. Reproductive state transitions: For example, PtoB (Prebreeder:With‐Pup), BtoJ (With‐Pup:With‐Juvenile), BtoN (With‐Pup:No‐Dependent). New = new cohorts (born 2001–2005), old = old cohorts (born 1994–1995). bs(Age) = basis spline fit to Age (df = 3), Age2 = quadratic fit to Age, Age = linear fit to Age. Age in PtoB: For example, 3, 4, 5p = age 3, 4, 5+ estimated separately. Cohort = year of birth for 2001–2005 cohorts (*n* = 5). Survival model: ac = annual survival for six age‐classes: 0–1, 1–2, 2–3, 3–15, 16–17, 18+; y1415 = years 2014–2015, y16 = year 2016; prime = prime ages 3–15, bad = bad years 2014–2016, a13 = after 2013, ac:B versus J = three age categories, for B: <7 (young), 7–15 (prime), 16+ (older), for J: <8 (young), 8–15 (prime), 16 + (older). W = White Sisters, V = Graves Rocks. Best models in sequence are in red (ΔAIC comparing best model to last best model).

With global models:

Offspring detection model = nr2:B + J

Survival model = ac + W + V + y1415 + y16

Transition model = move:breedgrp + PtoB:old + PtoB:new:Reg:3, 4, 5p + BtoN + BtoJ + NtoB + JtoN + JtoB

**Table:**

Model#	Model description	AIC	np	ΔAIC
(1) Female resighting rate model				
1	nr*grp + H:yr + R:yr + sb:grp (global)	666.2	93	
*Pool groups in term: nr*grp*				
2	ARP = ARN	671.7	88	
3	ARN = ARJ	669.9	88	
4	ARP = VR (ARPV)	656.8	88	
5	AHNP = AHBJ	670.0	92	
*Pool nr into regions in term: nr*grp*				
6	Reg:ARB	661.3	86	
7	Reg:ARN + nr:ARB	654.1	86	
8	Reg:ARPV + nr:ARB + Reg:ARN	651.3	84	
9	Reg:ARPV + nr:ARB + Reg:ARN + Reg:ARJ	648.1	82	
*Remove region effect in term: nr*grp*				
10	no Reg in ARN	646.3	81	
11	no Reg in ARPV or ARN	670.9	80	
12	no Reg in ARJ or ARN	651.5	80	
*Effect of sb in groups at rookeries*				
13	no ARB:sb *(Best)*	645.0	80	
14	no ARN:sb	648.4	80	
15	no ARPV:sb	668.2	80	
16	no ARJ:sb	829.9	80	
*Year effects*				
17	just R—no R*yr	722.6	66	
18	pooled yrs for H: 05, 06–14, 15+	676.5	72	
(2) Offspring detection model				
19	nr2:B + nr2:J	648.8	82	
20	nr:B + J	646.4	81	
21	B + J (no nr effect)	672.4	78	
22	constant model (B = J)	693.2	77	
(3) Transition model: movement				
23	ARP = ARN	656.6	78	
24	ARN = ARJ	670.3	78	
25	ARP = VR	711.0	78	
(4) Transition model: reproductive state				
26	PtoB—no Reg	669.0	77	
27	PtoB: S‐3,4,5p N‐3,4p	643.2	79	
28	PtoB: S‐3,4p N‐3,4p	648.5	78	
29	PtoB: 3‐Reg, 4pS, 5pS = 4pN	641.3	78	−3.6 vs. M13
30	PtoB: 3‐Reg, 4/5/6p‐S, 4/5p‐N	647.0	81	
31	BtoJ: bs(Age)	635.2	81	
32	BtoJ: bs(Age) + Reg	629.5	82	
33	BtoJ: bs(Age)*Reg	631.6	85	
34	BtoJ: Age2	633.4	80	
35	BtoJ: Age2 + Reg	627.6	81	−13.7 vs. M29
36	BtoJ: Age2*Reg	628.4	83	
37	BtoJ: Age	643.3	79	
38	BtoJ: Age + Reg	636.8	80	
39	BtoJ: Age*Reg	638.7	81	
40	BtoJ: Reg	635.5	79	
41	BtoN: bs(Age)	619.5	84	
42	BtoN: bs(Age) + Reg	621.4	85	
43	BtoN: bs(Age)*Reg	625.9	88	
44	BtoN: Age2	620.8	83	−6.8 vs. M35
45	BtoN: Age2 + Reg	622.8	84	
46	BtoN: Age2*Reg	622.3	86	
47	BtoN: Age	628.7	82	
48	BtoN: Age + Reg	629.9	83	
49	BtoN: Age*Reg	624.6	84	
50	BtoN: Reg	629.5	82	
51	JtoB: bs(Age)	625.2	86	
52	JtoB: bs(Age) + Reg	625.3	87	
53	JtoB: bs(Age) * Reg	629.7	90	
54	JtoB: Age2	623.4	85	
55	JtoB: Age2 + Reg	623.5	86	
56	JtoB: Age2*Reg	626.1	88	
57	JtoB: Age	621.6	84	
58	JtoB: Age + Reg	621.7	85	
59	JtoB: Age*Reg	623.6	86	
60	JtoB: Reg	621.4	84	
61	JtoN: bs(Age)	626.2	86	
62	JtoN: bs(Age) + Reg	627.3	87	
63	JtoN: bs(Age)*Reg	631.5	90	
64	JtoN: Age2	624.3	85	
65	JtoN: Age2 + Reg	625.3	86	
66	JtoN: Age2*Reg	628.6	88	
67	JtoN: Age	622.7	84	
68	JtoN: Age + Reg	623.8	85	
69	JtoN: Age*Reg	625.7	86	
70	JtoN: Reg	621.8	84	
71	NtoB: Reg	622.7	84	
72	BtoJ:yr *(Best)*	591.6	92	−29.2 vs. M44
73	+ BtoN:yr	590.6	101	
74	+ JtoB:yr	600.8	101	
75	+ JtoN:yr	602.1	101	
76	+ NtoB:yr	602.3	101	
77	cohort: PtoB	592.6	96	
78	cohort: BtoJ	596.7	97	
(5) Survival model				
79	ac + W + V + y1415:prime + y16:prime *(Best)*	586.5	92	−5.1 vs. M72
80	+ J + B	589.1	94	
81	+ J	588.4	93	
82	+ B	587.3	93	
83	+ N	588.5	93	
84	+ ac:B vs. J	592.6	98	
85	+ young:B vs. J	589.7	94	
86	+ older:B vs. J	587.4	94	
87	+ prime:BJ + older:BJ	586.9	94	
88	+ prime:N + older:N	587.3	94	
89	+ y1415:prime:J + y16:prime:J	602.4	92	
90	+ y1415:prime:BJ + y16:prime:BJ	587.0	92	
91	+ y1415:prime + y16:prime:J	599.1	92	
92	+ y1415:prime + y16:prime:BJ	586.5	92	
93	+ y1415:prime + y16:prime + J:bad	587.1	93	
94	+ y1415:prime + y16:prime + BJ:bad	586.2	93	
95	+ y1415:prime + y16:prime + J:a13	588.4	93	
96	+ y1415:prime + y16:prime + BJ:a13	588.4	93	
97	+ y1415:prime:N + y16:prime:N	604.3	92	
98	+ y1415:prime + y16:prime:N	596.3	92	
99	+ y1415:prime + y16:prime + N:bad	588.2	93	
100	+ y1415:prime + y16:prime + N:a13	587.5	93	
101	+ y1415:prime:B + y16:prime:B	589.1	92	
102	+ y1415:prime + y16:prime + B:bad	588.4	93	
103	+ y1415:prime + y16:prime + B:a13	588.4	93	

## References

[ece310515-bib-0001] Alaska Fisheries Science Center . (2023). Alaska Steller sea lion pup count database . https://www.fisheries.noaa.gov/inport/item/24573

[ece310515-bib-0002] Albon, S. D. , Coulson, T. N. , Brown, D. , Guinness, F. E. , Pemberton, J. M. , & Clutton‐Brock, T. H. (2000). Temporal changes in key factors and key age groups influencing the population dynamics of female red deer. Journal of Animal Ecology, 69, 1099–1110.

[ece310515-bib-0003] Albouy, C. , Delattre, V. , Donati, G. , Frölicher, T. L. , Albouy‐Boyer, S. , Rufino, M. , Pellissier, L. , Mouillet, D. , & Leprieur, F. (2020). Global vulnerability of marine mammals to global warming. Nature Scientific Reports, 10, 548. 10.1038/s41598-019-57280-3 PMC6969058 31953496

[ece310515-bib-0004] Altukhov, A. V. , Andrews, R. D. , Calkins, D. G. , Gelatt, T. S. , Gurarie, E. D. , Loughlin, T. R. , Mamaev, E. G. , Nikulin, V. S. , Permyakov, P. A. , Ryazanov, S. D. , Vertyankin, V. V. , & Burkanov, V. N. (2015). Age specific survival rates of Steller sea lions at rookeries with divergent population trends in the Russian Far East. PLoS One, 10(5), e0127292. 26016772 10.1371/journal.pone.0127292 PMC4446299

[ece310515-bib-0005] Arimitsu, M. L. , Piatt, J. F. , Hatch, S. , Suryan, R. M. , Batten, S. , Bishop, M. A. , Campbell, R. W. , Coletti, H. , Cushing, D. , Gorman, K. , Hopcroft, R. R. , Kuletz, K. J. , Marsteller, C. , McKinstry, C. , McGowan, D. , Moran, J. , Pegau, S. , Schaefer, A. , Schoen, S. , … von Biela, V. R. (2021). Heatwave‐induced synchrony within forage fish portfolio disrupts energy flow to top pelagic predators. Global Change Biology, 27, 1859–1878. 33577102 10.1111/gcb.15556 PMC8048560

[ece310515-bib-0006] Beauplet, G. , Barbraud, C. , Dabin, W. , Küssener, C. , & Guinet, C. (2006). Age‐specific survival and reproductive performances in fur seals: Evidence of senescence and individual quality. Oikos, 112, 430–441.

[ece310515-bib-0007] Boltnev, A. I. , & York, A. E. (2001). Maternal investment in northern fur seals (*Callorhinus ursinus*): Interrelationships among mothers' age, size, parturition date, offspring size and sex ratios. Journal of Zoology (London), 254, 219–228.

[ece310515-bib-0008] Bond, N. A. , Cronin, M. F. , Freeland, H. , & Mantua, N. (2015). Causes and impacts of the 2014 warm anomaly in the NE Pacific. Geophysical Research Letters, 42, 3414–3420.

[ece310515-bib-0009] Bonner, W. N. (1984). Lactation strategies in pinnipeds: Problems for a marine mammalian group. Symposia of the Zoological Society of London, 51, 253–272.

[ece310515-bib-0010] Bouwhuis, S. , Choquet, R. , Sheldon, B. C. , & Verhulst, S. (2012). The forms and fitness cost of senescence: Age‐specific recapture, survival, reproduction, and reproductive value in a wild bird population. American Naturalist, 179, E15–E27. 10.1086/663194 22173469

[ece310515-bib-0011] Bowles, M. L. , McBride, J. L. , & Bell, T. J. (2015). Long‐term processes affecting restoration and viability of the federal threatened Mead's milkweed (*Asclepias meadii*). Ecosphere, 6(1), 11–22.

[ece310515-bib-0012] Boyd, I. L. , Croxall, J. P. , Lunn, N. J. , & Reid, K. (1995). Population demography of Antarctic fur seals: The costs of reproduction and implications for life‐histories. Journal of Animal Ecology, 64, 505–518.

[ece310515-bib-0013] Bronson, F. H. (1985). Mammalian reproduction: An ecological perspective. Biology of Reproduction, 32, 1–26. 3882162 10.1095/biolreprod32.1.1

[ece310515-bib-0014] Burnham, K. P. , & Anderson, D. R. (2002). Model selection and inference: A practical information‐theoretic approach (2nd ed.). Springer.

[ece310515-bib-0015] Calkins, D. , & Goodwin, E. (1988). Investigation of the declining sea lion population in the Gulf of Alaska . August 15, 1988 (pp. 76). Technical report of the Alaska Department of Fish and Game.

[ece310515-bib-0016] Calkins, D. G. , Becker, E. F. , & Pitcher, K. W. (1998). Reduced body size of female Steller sea lions from a declining population in the Gulf of Alaska. Marine Mammal Science, 14, 232–244.

[ece310515-bib-0017] Chambert, T. , Rotella, J. J. , Higgs, M. D. , & Garrott, R. A. (2013). Individual heterogeneity in reproductive rates and cost of reproduction in a long‐lived vertebrate. Ecology and Evolution, 3, 2047–2060. 23919151 10.1002/ece3.615 PMC3728946

[ece310515-bib-0018] Chen, Z. , Shi, J. , Liu, Q. , Chen, H. , & Li, C. (2021). A persistent and intense marine heatwave in the northeast pacific during 2019–2020. Geophysical Research Letters, 48, e2021GL093239.

[ece310515-bib-0019] Childerhouse, S. J. , Dawson, S. M. , Fletcher, D. J. , Slooten, E. , & Chilvers, B. L. (2010). Growth and reproduction of female New Zealand sea lions. Journal of Mammalogy, 91, 165–176.

[ece310515-bib-0020] Colchero, F. , Jones, O. R. , Conde, D. A. , Hodgson, D. , Zajitschek, F. , Schmidt, B. R. , Malo, A. F. , Alberts, S. C. , Becker, P. H. , Bouwhuis, S. , Bronikowski, A. M. , Vleeschouwer, K. M. , Delahay, R. J. , Dummermuth, S. , Fernández‐Duque, E. , Frisenvænge, J. , Hesselsøe, M. , Larson, S. , Lemaître, J. F. , … Gaillard, J. M. (2019). The diversity of population responses to environmental change. Ecology Letters, 22, 342–353. 30536594 10.1111/ele.13195 PMC6378614

[ece310515-bib-0021] Cole, L. C. (1954). The population consequences of life history phenomena. Quarterly Review of Biology, 29, 103–137. 13177850 10.1086/400074

[ece310515-bib-0022] Comizzoli, P. , & Ottinger, M. A. (2021). Understanding reproductive aging in wildlife to improve animal conservation and human reproductive health. Frontiers in Cell and Developmental Biology, 9, 680471. 34095152 10.3389/fcell.2021.680471 PMC8170016

[ece310515-bib-0023] Costa, D. P. , & Valenzuela‐Toro, A. M. (2021). When physiology and ecology meet: The interdependency between foraging ecology and reproduction in otariids. In C. Campagna & R. Harcourt (Eds.), Ethology and behavioral ecology of Otariids and the Odobenid, ethology and behavioral ecology of marine mammals (pp. 21–50). Springer.

[ece310515-bib-0024] Coulson, T. , Gaillard, J.‐M. , & Festa‐Bianchet, M. (2005). Decomposing the variation in population growth into contributions from multiple demographic rates. Journal of Animal Ecology, 74, 789–801.

[ece310515-bib-0025] Coulson, T. , & Hudson, E. (2003). When is birth rate the key factor associated with population dynamics? In W. V. Holt , A. R. Pickard , J. C. Rodger , & D. E. Wildt (Eds.), Reproductive science and integrated conservation (pp. 114–128). Cambridge University Press.

[ece310515-bib-0026] Dabin, W. , Beauplet, G. , Crespo, E. A. , & Guinet, C. (2004). Age structure, growth, and demographic parameters in breeding‐age female subantarctic fur seals, *Arctocephalus tropicalis* . Canadian Journal of Zoology, 82, 1043–1050.

[ece310515-bib-0027] Danielson, S. L. , Hennon, T. D. , Monson, D. H. , Suryan, R. M. , Campbell, R. W. , Baird, S. J. , Holderied, K. , & Weingartner, T. J. (2022). Temperature variations in the northern Gulf of Alaska across synoptic to century‐long time scales. Deep‐Sea Research Part II, 203, 105155.

[ece310515-bib-0028] Doak, D. F. , Morris, W. F. , Pfister, C. , Kendall, B. E. , & Bruna, E. M. (2005). Correctly estimating how environmental stochasticity influences fitness and population growth. The American Naturalist, 66, E14–E21. 10.1086/430642 15937784

[ece310515-bib-0029] Eberhardt, L. L. (1985). Assessing the dynamics of wild populations. Journal of Wildlife Management, 49, 997–1012.

[ece310515-bib-0030] Edie, A. G. (1977). Distribution and movements of Steller sea lion cows (Eumetopias jubatus) on a pupping colony (M.S. thesis). University of British Columbia.

[ece310515-bib-0031] Fowler, C. W. (1987). A review of density dependence in populations of large mammals. In H. H. Genoways (Ed.), Current mammalogy (Vol. 1, pp. 401–441). Plenum Press.

[ece310515-bib-0032] Fritz, L. , Sweeney, K. , Towell, R. , & Gelatt, T. (2016). *Aerial and ship‐based surveys of Steller sea lions (*Eumetopias jubatus*) conducted in Alaska in June–July 2013 through 2015, and an update on the status and trend of the western distinct population segment in Alaska* . US Department of Commerce, NOAA Technical Memorandum NMFS‐AFSC‐321.

[ece310515-bib-0033] Fritz, L. W. , Towell, R. , Gelatt, T. S. , Johnson, D. S. , & Loughlin, T. R. (2014). Recent increases in survival of western Steller sea lions in Alaska and implications for recovery. Endangered Species Research, 26, 13–24.

[ece310515-bib-0034] Gaillard, J.‐M. , Festa‐Bianchet, M. , Yoccoz, N. G. , Loison, A. , & Toïgo, T. (2000). Temporal variation in fitness components and population dynamics of large herbivores. Annual Review of Ecology, Evolution, and Systematics, 31, 367–393.

[ece310515-bib-0035] Grandi, M. F. , Dans, S. L. , García, N. A. , & Crespo, E. A. (2010). Growth and age at sexual maturity of South American sea lions. Mammalian Biology, 75, 427–436.

[ece310515-bib-0036] Hamel, S. , Côte, S. D. , Gaillard, J.‐M. , & Festa‐Bianchet, M. (2009). Individual variation in reproductive costs of reproduction: High‐quality females always do better. Journal of Animal Ecology, 78, 143–151. 18700872 10.1111/j.1365-2656.2008.01459.x

[ece310515-bib-0037] Hastings, K. K. (2017). Survival of Steller sea lion (*Eumetopias jubatus*) pups during the first months of life at the Forrester Island complex, Alaska. Journal of Mammalogy, 98, 397–409.

[ece310515-bib-0038] Hastings, K. K. , Gelatt, T. S. , & King, J. C. (2009). Postbranding survival of Steller sea lion pups at Lowrie Island in Southeast Alaska. Journal of Wildlife Management, 73, 1040–1051.

[ece310515-bib-0039] Hastings, K. K. , Gelatt, T. S. , Maniscalco, J. M. , Jemison, L. A. , Towell, R. , Pendleton, G. W. , & Johnson, D. S. (2023). Reduced survival of Steller sea lions in the Gulf of Alaska following marine heatwave. Frontiers in Marine Science, 10, 1127013.

[ece310515-bib-0040] Hastings, K. K. , & Jemison, L. A. (2016). Age‐specific variation in timing of parturition in Steller sea lions at Forrester Island complex, Alaska. Marine Mammal Science, 32, 777–785.

[ece310515-bib-0041] Hastings, K. K. , Jemison, L. A. , Gelatt, T. S. , Laake, J. L. , Pendleton, G. W. , King, J. C. , Trites, A. W. , & Pitcher, K. W. (2011). Cohort effects and spatial variation in age‐specific survival of Steller sea lions from southeastern Alaska. Ecosphere, 2, 111.

[ece310515-bib-0042] Hastings, K. K. , Jemison, L. A. , & Pendleton, G. W. (2018). Survival of adult Steller sea lions in Alaska: Senescence, annual variation and covariation with male reproductive success. Royal Society Open Science, 5, 170665. 29410794 10.1098/rsos.170665 PMC5792871

[ece310515-bib-0043] Hastings, K. K. , Johnson, D. S. , & Gelatt, T. S. (2017). Flipper tag loss in Steller sea lions. Marine Mammal Science, 34, 229–237.

[ece310515-bib-0044] Hastings, K. K. , Johnson, D. S. , Pendleton, G. W. , Fadely, B. S. , & Gelatt, T. S. (2021). Investigating life‐ history traits of Steller sea lions with multistate hidden Markov mark‐recapture models: Age at weaning and body size effects. Ecology and Evolution, 11, 714–734. 33520160 10.1002/ece3.6878 PMC7820167

[ece310515-bib-0045] Hennemann, W. W. (1983). Relationship among body mass, metabolic rate and the intrinsic rate of natural increase in mammals. Oecologia, 56, 104–108. 28310776 10.1007/BF00378224

[ece310515-bib-0046] Hernández‐Camacho, C. J. , Aurioles‐Gamboa, D. , & Gerber, L. R. (2008). Age‐specific birth rates of California sea lions (*Zalophus californianus*) in the Gulf of California, Mexico. Marine Mammal Science, 24, 664–676.

[ece310515-bib-0047] Jemison, L. A. , Pendleton, G. W. , Fritz, L. W. , Hastings, K. K. , Maniscalco, J. M. , Trites, A. W. , & Gelatt, T. S. (2013). Inter‐population movements of Steller sea lions in Alaska with implications for population separation. PLoS One, 8, e70167. 23940543 10.1371/journal.pone.0070167 PMC3734025

[ece310515-bib-0048] Jemison, L. A. , Pendleton, G. W. , Hastings, K. K. , Maniscalco, J. M. , & Fritz, L. W. (2018). Spatial distribution, movements, and geographic range of Steller sea lions (*Eumetopias jubatus*) in Alaska. PLoS One, 13, e0208093. 30586412 10.1371/journal.pone.0208093 PMC6306159

[ece310515-bib-0049] Johnson, D. S. , Laake, J. L. , Melin, S. R. , & DeLong, R. L. (2016). Multivariate state hidden Markov models for mark‐recapture data. Statistical Science, 31, 233–244.

[ece310515-bib-0050] Kalberer, S. , Meise, K. , Trillmich, F. , & Krüger, O. (2018). Reproductive performance of a tropical apex predator in an unpredictable habitat. Behavioral Ecology and Sociobiology, 72, 108.

[ece310515-bib-0051] Kaplan, C. C. , White, G. C. , & Noon, B. R. (2008). Neonatal survival of Steller sea lions (*Eumetopias jubatus*). Marine Mammal Science, 24, 443–461.

[ece310515-bib-0052] King, J. E. (1983). Seals of the world (2nd ed.). Cornell University Press.

[ece310515-bib-0053] Kuhn, C. E. , Chumbley, K. , Johnson, D. , & Fritz, L. (2017). A re‐examination of the timing of pupping for Steller sea lions *Eumetopias jubatus* breeding on two islands in Alaska. Endangered Species Research, 32, 213–222.

[ece310515-bib-0054] Laake, J. L. , Johnson, D. S. , & Conn, P. B. (2013). Marked: An R package for maximum likelihood and Markov chain Monte Carlo analysis of capture–recapture data. Methods in Ecology and Evolution, 4, 885–890.

[ece310515-bib-0055] Laake, J. L. , Johnson, D. S. , Diefenbach, D. R. , & Ternent, M. A. (2014). Hidden Markov model for dependent mark loss and survival estimation. Journal of Agricultural, Biological, and Environmental Statistics, 19, 524–540.

[ece310515-bib-0056] Laws, R. M. (1956). Growth and sexual maturity in aquatic mammals. Nature, 178, 193–194.

[ece310515-bib-0057] Le Boeuf, B. , Condit, R. , & Reiter, J. (2019). Lifetime reproductive success of northern elephant seals (*Mirounga angustirostris*). Canadian Journal of Zoology, 97, 1203–1217.

[ece310515-bib-0058] Lebreton, J.‐D. , Burnham, K. P. , Clobert, J. , & Anderson, D. R. (1992). Modeling survival and testing biological hypotheses using marked animals: A unified approach with case studies. Ecological Monographs, 62, 67–118.

[ece310515-bib-0059] Lunn, N. J. , Boyd, I. L. , & Croxall, J. P. (1994). Reproductive performance of female Antarctic fur seals: The influence of age, breeding experience, environmental variation and individual quality. Journal of Animal Ecology, 63, 827–840.

[ece310515-bib-0060] Maniscalco, J. M. (2014). The effects of birth weight and maternal care on survival of juvenile Steller sea lions (*Eumetopias jubatus*). PLoS One, 9, e96328. 24804679 10.1371/journal.pone.0096328 PMC4012995

[ece310515-bib-0061] Maniscalco, J. M. , Calkins, D. G. , Parker, P. , & Atkinson, S. (2008). Causes and extent of natural mortality among Steller sea lion (*Eumetopias jubatus*) pups. Aquatic Mammals, 34, 277–287.

[ece310515-bib-0062] Maniscalco, J. M. , & Parker, P. (2009). A case of twinning and the care of two offspring of different age in Steller sea lions. Marine Mammal Science, 25, 206–213.

[ece310515-bib-0063] Maniscalco, J. M. , & Parker, P. (2018). Maternal and offspring effects on the timing of parturition in western Steller sea lions (*Eumetopias jubatus*). Canadian Journal of Zoology, 96, 333–339.

[ece310515-bib-0064] Maniscalco, J. M. , Springer, A. M. , Parker, P. , & Adkison, M. D. (2014). A longitudinal study of Steller sea lion natality rates in the Gulf of Alaska with comparisons to census data. PLoS One, 9, e111523. 25383865 10.1371/journal.pone.0111523 PMC4226517

[ece310515-bib-0065] Manlik, O. , McDonald, J. A. , Mann, J. , Raudino, H. C. , Bejder, L. , Krützen, M. , Connor, R. C. , Heithaus, M. R. , Lacy, R. C. , & Sherwin, W. B. (2016). The relative importance of reproduction and survival for the conservation of two dolphin populations. Ecology and Evolution, 6, 3496–3512. 28725349 10.1002/ece3.2130 PMC5513288

[ece310515-bib-0066] Mathews, E. A. , Womble, J. N. , Pendleton, G. W. , Jemison, L. A. , Maniscalco, J. M. , & Streveler, G. (2011). Population growth and colonization of Steller sea lions in the Glacier Bay region of southeastern Alaska: 1970s–2009. Marine Mammal Science, 27, 852–880.

[ece310515-bib-0067] McKenzie, J. , Page, B. , Shaughnessy, P. D. , & Hindell, M. A. (2007). Age and reproductive maturity of New Zealand fur seals (*Arctocephalus forsteri*) in southern Australia. Journal of Mammalogy, 88, 639–648.

[ece310515-bib-0068] McMahon, C. R. , Burton, H. R. , Van Den Hoff, J. , Woods, R. , & Bradshaw, C. J. A. (2006). Assessing hot‐iron and cryo‐branding for permanently marking southern elephant seals. Journal of Wildlife Management, 70, 1484–1489.

[ece310515-bib-0069] Melin, S. R. , Laake, J. L. , DeLong, R. L. , & Siniff, D. B. (2012). Age‐specific recruitment and natality of California sea lions at San Miguel Island, California. Marine Mammal Science, 28, 751–776.

[ece310515-bib-0070] Merrick, R. L. , Loughlin, T. R. , & Calkins, D. G. (1996). Hotbranding: A technique for long‐term marking of pinnipeds . U.S. National Oceanic and Atmospheric Administration technical memorandum NMFSAFSC‐68.

[ece310515-bib-0071] NMFS . (2008). Recovery plan for the Steller Sea lion (Eumetopias jubatus). Revision. National Marine Fisheries Service.

[ece310515-bib-0072] Oliver, E. C. J. , Donat, M. G. , Burrows, M. T. , Moore, P. J. , Smale, D. A. , Alexander, L. V. , Benthuysen, J. A. , Feng, M. , Sen Gupta, A. , Hobday, A. J. , Holbrook, N. J. , Perkins‐Kirkpatrick, S. E. , Scannell, H. A. , Straub, S. C. , & Wernberg, T. (2018). Longer and more frequent marine heatwaves over the past century. Nature Communications, 9, 1324. 10.1038/s41467-018-03732-9 PMC5893591 29636482

[ece310515-bib-0073] Ottinger, M. A. (2010). Mechanisms of reproductive aging: Conserved mechanisms and environmental factors. Annals of the New York Academy of Sciences, 1204, 73–81. 20738277 10.1111/j.1749-6632.2010.05653.x PMC2929979

[ece310515-bib-0074] Pendleton, G. W. , Pitcher, K. W. , Fritz, L. W. , York, A. E. , Raum‐Suryan, K. L. , Loughlin, T. R. , Calkins, D. G. , Hastings, K. K. , & Gelatt, T. S. (2006). Survival of Steller sea lions in Alaska: A comparison of increasing and decreasing populations. Canadian Journal of Zoology, 84, 1163–1172.

[ece310515-bib-0075] Pitcher, K. W. , Burkanov, V. N. , Calkins, D. G. , Le Boeuf, B. J. , Mamaev, E. G. , Merrick, R. L. , & Pendleton, G. W. (2001). Spatial and temporal variation in the timing of births of Steller sea lions. Journal of Mammalogy, 82, 1047–1053.

[ece310515-bib-0076] Pitcher, K. W. , & Calkins, D. G. (1981). Reproductive biology of Steller sea lions in the Gulf of Alaska. Journal of Mammalogy, 62, 599–605.

[ece310515-bib-0077] Pitcher, K. W. , Calkins, D. G. , & Pendleton, G. W. (1998). Reproductive performance of female Steller sea lions: An energetics‐based reproductive strategy? Canadian Journal of Zoology, 76, 2075–2083.

[ece310515-bib-0078] Pitcher, K. W. , Olesiuk, P. F. , Brown, R. F. , Lowry, M. S. , Jeffries, S. J. , Sease, J. L. , Perryman, W. L. , Stinchcomb, C. E. , & Lowry, L. F. (2007). Abundance and distribution of the eastern North Pacific Steller sea lion (*Eumetopias jubatus*) population. Fishery Bulletin, 107, 102–115.

[ece310515-bib-0079] Pollock, K. H. (1982). A capture‐recapture design robust to unequal probability of capture. Journal of Wildlife Management, 46, 752–757.

[ece310515-bib-0080] R Core Team . (2022). R: A language and environment for statistical computing. R Foundation of Statistical Computing. https://www.R‐project.org/

[ece310515-bib-0081] Rehberg, M. , Jemison, L. , Womble, J. N. , & O'Corry‐Crowe, G. (2018). Winter movements and long‐term dispersal of Steller sea lions in the Glacier Bay region of Southeast Alaska. Endangered Species Research, 37, 11–24.

[ece310515-bib-0082] Sandegren, F. E. (1970). *Breeding and maternal behavior of the Steller sea lion (*Eumetopias jubatus*) in Alaska* (Master's thesis). University of Alaska.

[ece310515-bib-0083] Schwarz, L. K. , Goebel, M. E. , Costa, D. P. , & Kilpatrick, A. M. (2013). Top‐down and bottom‐up influences on demographic rates of Antarctic fur seals *Arctocephalus gazella* . Journal of Animal Ecology, 82, 903–911. 23444975 10.1111/1365-2656.12059

[ece310515-bib-0084] Skalski, J. R. , Millspaugh, J. J. , Dillingham, P. , & Buchanan, R. A. (2007). Calculating the variance of the finite rate of population change from a matrix model in Mathematica. Environmental Modelling & Software, 22, 359–364.

[ece310515-bib-0085] Stearns, S. C. (1976). Life‐history tactics: A review of the ideas. The Quarterly Review of Biology, 51, 3–47. 778893 10.1086/409052

[ece310515-bib-0086] Stearns, S. C. (1989). Trade‐offs in life‐history evolution. Functional Ecology, 3, 259–268.

[ece310515-bib-0087] Stubben, C. J. , & Milligan, B. G. (2007). Estimating and analyzing demographic models using the popbio package in R. Journal of Statistical Software, 22, 11.

[ece310515-bib-0088] Suryan, R. M. , Arimitsu, M. L. , Coletti, H. A. , Hopcroft, R. R. , Lindeberg, M. R. , Barbeaux, S. J. , Batten, S. D. , Burt, W. J. , Bishop, M. A. , Bodkin, J. L. , Brenner, R. , Campbell, R. W. , Cushing, D. A. , Danielson, S. L. , Dorn, M. W. , Drummond, B. , Esler, D. , Gelatt, T. , Hanselman, D. H. , … Zador, S. G. (2021). Ecosystem response persists after a prolonged marine heatwave. Nature Scientific Reports, 11, 6235. 10.1038/s41598-021-83818-5 PMC7973763 33737519

[ece310515-bib-0089] Taylor, R. L. , & Boor, G. K. H. (2012). Beyond the robust design: Accounting for changing, uncertain states and sparse, biased detection in a multistate mark‐recapture model. Ecological Modelling, 243, 73–80.

[ece310515-bib-0090] Wade, G. N. , & Schneider, J. E. (1992). Metabolic fuels and reproduction in female mammals. Neuroscience & Biobehavioral Reviews, 16, 235–272. 1630733 10.1016/s0149-7634(05)80183-6

[ece310515-bib-0091] Warlick, A. J. , Johnson, D. S. , Gelatt, T. S. , & Converse, S. J. (2022). Environmental drivers of demography and potential factors limiting the recovery of an endangered marine top predator. Ecosphere, 13, e4325.

[ece310515-bib-0092] Winship, A. J. , Trites, A. W. , & Calkins, D. G. (2001). Growth in body size of the Steller sea lion (*Eumetopias jubatus*). Journal of Mammalogy, 82, 500–519.

[ece310515-bib-0093] Wright, B. E. , Brown, R. F. , DeLong, R. L. , Gearin, P. J. , Riemer, S. D. , Laake, J. L. , & Scordino, J. J. (2017). Survival rates of Steller sea lions from Oregon and California. Journal of Mammalogy, 98, 885–894.

[ece310515-bib-0094] York, A. E. (1994). The population dynamics of northern sea lions, 1975–1985. Marine Mammal Science, 10, 38–51.

[ece310515-bib-0095] York, A. E. , Merrick, R. L. , & Loughlin, T. R. (1996). An analysis of the Steller sea lion metapopulation in Alaska. In D. R. McCullough (Ed.), Metapopulations and wildlife conservation (pp. 259–292). Island Press.

