# Supplementary material for: Age‐specific reproduction in female Steller sea lions in Southeast Alaska
Source: Ecol Evol. 2023 Sep 27;13(9):e10515. doi: 10.1002/ece3.10515 (PMC10533480; doi:10.1002/ece3.10515)
Supplement: Supplementary file 1 — Table S1. Table S2. Table S3. [file ECE3-13-e10515-s001.docx]

Supplemental Table S1. Estimates of the proportions of Steller sea lion females in Southeast Alaska (2005–2019) by reproductive state at the end of the pupping season, age and natal region. Natal regions were South (Forrester and Hazy) and North (White Sisters and Graves Rocks, Figure 1). 95% CI are in parentheses. Estimates are for years < 2011, before the Pacific Marine Heatwave; data are plotted in Figure 2. The last two columns are estimates of early pup survival (birth to age 3 weeks) by maternal age for pups born at Forrester Islands in 2007.

| Age | North | | | | | | | | South | | | | | | | | Mom Age | Pup Survival |
| --- | --- | --- | --- | --- | --- | --- | --- | --- | --- | --- | --- | --- | --- | --- | --- | --- | --- | --- |
|  | Prebreeder | | With-Pup | | With-Juvenile | | No-Dependent | | Prebreeder | | With-Pup | | With-Juvenile | | No-Dependent | |  |  |
| 1 | 1 | | 0 | | 0 | | 0 | | 1 | | 0 | | 0 | | 0 | |  |  |
| 2 | 1 | | 0 | | 0 | | 0 | | 1 | | 0 | | 0 | | 0 | |  |  |
| 3 | 1 | | 0 | | 0 | | 0 | | 1 | | 0 | | 0 | | 0 | |  |  |
| 4 | 0.900 | *( 0.799 - 0.952 )* | 0.100 | *( 0.048 - 0.201 )* | 0 | | 0 | | 1 | | 0 | | 0 | | 0 | |  |  |
| 5 | 0.436 | *( 0.342 - 0.528 )* | 0.487 | *( 0.404 - 0.567 )* | 0.063 | *( 0.028 - 0.125 )* | 0.014 | *( 0.004 - 0.045 )* | 0.736 | *( 0.630 - 0.819 )* | 0.264 | *( 0.181 - 0.370 )* | 0 | | 0 | | 5 | 0.755 |
| 6 | 0.211 | *( 0.137 - 0.301 )* | 0.420 | *( 0.368 - 0.480 )* | 0.301 | *( 0.229 - 0.369 )* | 0.067 | *( 0.034 - 0.128 )* | 0.357 | *( 0.271 - 0.442 )* | 0.480 | *( 0.411 - 0.547 )* | 0.128 | *( 0.083 - 0.183 )* | 0.035 | *( 0.014 - 0.077 )* | 6 | 0.780 |
| 7 | 0.102 | *( 0.055 - 0.173 )* | 0.478 | *( 0.422 - 0.532 )* | 0.318 | *( 0.259 - 0.374 )* | 0.102 | *( 0.065 - 0.154 )* | 0.173 | *( 0.110 - 0.251 )* | 0.501 | *( 0.446 - 0.555 )* | 0.248 | *( 0.197 - 0.300 )* | 0.077 | *( 0.047 - 0.124 )* | 7 | 0.804 |
| 8 | 0.050 | *( 0.022 - 0.099 )* | 0.516 | *( 0.457 - 0.572 )* | 0.328 | *( 0.268 - 0.388 )* | 0.106 | *( 0.071 - 0.158 )* | 0.084 | *( 0.044 - 0.143 )* | 0.548 | *( 0.491 - 0.599 )* | 0.271 | *( 0.222 - 0.322 )* | 0.097 | *( 0.065 - 0.144 )* | 8 | 0.825 |
| 9 | 0.024 | *( 0.009 - 0.057 )* | 0.545 | *( 0.481 - 0.605 )* | 0.326 | *( 0.264 - 0.388 )* | 0.105 | *( 0.070 - 0.157 )* | 0.041 | *( 0.017 - 0.082 )* | 0.587 | *( 0.527 - 0.639 )* | 0.274 | *( 0.224 - 0.326 )* | 0.099 | *( 0.067 - 0.148 )* | 9 | 0.844 |
| 10 | 0.012 | *( 0.003 - 0.033 )* | 0.568 | *( 0.498 - 0.634 )* | 0.319 | *( 0.253 - 0.384 )* | 0.101 | *( 0.068 - 0.154 )* | 0.020 | *( 0.007 - 0.047 )* | 0.615 | *( 0.551 - 0.669 )* | 0.269 | *( 0.219 - 0.324 )* | 0.096 | *( 0.064 - 0.146 )* | 10 | 0.862 |
| 11 | 0.006 | *( 0.001 - 0.019 )* | 0.584 | *( 0.508 - 0.656 )* | 0.312 | *( 0.244 - 0.381 )* | 0.098 | *( 0.065 - 0.151 )* | 0.010 | *( 0.003 - 0.027 )* | 0.633 | *( 0.566 - 0.691 )* | 0.264 | *( 0.212 - 0.320 )* | 0.093 | *( 0.061 - 0.144 )* | 11 | 0.877 |
| 12 | 0.003 | *( 0.001 - 0.011 )* | 0.592 | *( 0.513 - 0.668 )* | 0.310 | *( 0.237 - 0.380 )* | 0.096 | *( 0.062 - 0.149 )* | 0.005 | *( 0.001 - 0.016 )* | 0.643 | *( 0.572 - 0.704 )* | 0.261 | *( 0.207 - 0.319 )* | 0.091 | *( 0.059 - 0.144 )* | 12 | 0.891 |
| 13 | 0.001 | *( 0.000 - 0.006 )* | 0.592 | *( 0.509 - 0.672 )* | 0.312 | *( 0.236 - 0.384 )* | 0.095 | *( 0.061 - 0.151 )* | 0.002 | *( 0.000 - 0.009 )* | 0.644 | *( 0.571 - 0.709 )* | 0.263 | *( 0.206 - 0.323 )* | 0.091 | *( 0.058 - 0.147 )* | 13 | 0.904 |
| 14 | 0.001 | *( 0.000 - 0.004 )* | 0.583 | *( 0.498 - 0.667 )* | 0.319 | *( 0.241 - 0.393 )* | 0.097 | *( 0.063 - 0.155 )* | 0.001 | *( 0.000 - 0.005 )* | 0.635 | *( 0.559 - 0.703 )* | 0.271 | *( 0.211 - 0.332 )* | 0.093 | *( 0.060 - 0.153 )* | 14 | 0.915 |
| 15 | 0.000 | *( 0.000 - 0.002 )* | 0.566 | *( 0.481 - 0.652 )* | 0.332 | *( 0.249 - 0.406 )* | 0.102 | *( 0.066 - 0.163 )* | 0.001 | *( 0.000 - 0.003 )* | 0.617 | *( 0.538 - 0.688 )* | 0.284 | *( 0.220 - 0.347 )* | 0.099 | *( 0.063 - 0.163 )* | 15 | 0.925 |
| 16 | 0.000 | *( 0.000 - 0.001 )* | 0.541 | *( 0.460 - 0.630 )* | 0.349 | *( 0.261 - 0.425 )* | 0.110 | *( 0.071 - 0.175 )* | 0.000 | *( 0.000 - 0.002 )* | 0.590 | *( 0.510 - 0.667 )* | 0.302 | *( 0.233 - 0.368 )* | 0.109 | *( 0.069 - 0.178 )* | 16 | 0.933 |
| 17 | 0.000 | *( 0.000 - 0.001 )* | 0.511 | *( 0.435 - 0.601 )* | 0.368 | *( 0.277 - 0.444 )* | 0.121 | *( 0.078 - 0.190 )* | 0.000 | *( 0.000 - 0.001 )* | 0.555 | *( 0.476 - 0.639 )* | 0.323 | *( 0.248 - 0.392 )* | 0.122 | *( 0.078 - 0.197 )* | 17 | 0.941 |
| 18 | 0.000 | *( 0.000 - 0.000 )* | 0.479 | *( 0.408 - 0.569 )* | 0.387 | *( 0.291 - 0.463 )* | 0.134 | *( 0.088 - 0.209 )* | 0.000 | *( 0.000 - 0.001 )* | 0.516 | *( 0.440 - 0.607 )* | 0.344 | *( 0.262 - 0.416 )* | 0.140 | *( 0.089 - 0.221 )* | 18 | 0.948 |
| 19 | 0.000 | *( 0.000 - 0.000 )* | 0.448 | *( 0.386 - 0.536 )* | 0.401 | *( 0.299 - 0.476 )* | 0.151 | *( 0.100 - 0.233 )* | 0.000 | *( 0.000 - 0.000 )* | 0.477 | *( 0.408 - 0.571 )* | 0.361 | *( 0.269 - 0.436 )* | 0.162 | *( 0.104 - 0.249 )* | 19 | 0.954 |
| 20 | 0.000 | *( 0.000 - 0.000 )* | 0.423 | *( 0.369 - 0.505 )* | 0.407 | *( 0.297 - 0.483 )* | 0.170 | *( 0.112 - 0.262 )* | 0.000 | *( 0.000 - 0.000 )* | 0.444 | *( 0.385 - 0.534 )* | 0.369 | *( 0.266 - 0.448 )* | 0.187 | *( 0.122 - 0.282 )* | 20 | 0.960 |
| 21 | 0.000 | *( 0.000 - 0.000 )* | 0.406 | *( 0.357 - 0.477 )* | 0.403 | *( 0.279 - 0.486 )* | 0.192 | *( 0.125 - 0.300 )* | 0.000 | *( 0.000 - 0.000 )* | 0.421 | *( 0.370 - 0.500 )* | 0.365 | *( 0.248 - 0.453 )* | 0.214 | *( 0.138 - 0.324 )* |  |  |
| 22 | 0.000 | *( 0.000 - 0.000 )* | 0.396 | *( 0.350 - 0.455 )* | 0.389 | *( 0.239 - 0.483 )* | 0.216 | *( 0.134 - 0.344 )* | 0.000 | *( 0.000 - 0.000 )* | 0.407 | *( 0.360 - 0.472 )* | 0.348 | *( 0.208 - 0.453 )* | 0.244 | *( 0.152 - 0.373 )* |  |  |
| 23 | 0.000 | *( 0.000 - 0.000 )* | 0.392 | *( 0.346 - 0.444 )* | 0.364 | *( 0.186 - 0.482 )* | 0.244 | *( 0.142 - 0.396 )* | 0.000 | *( 0.000 - 0.000 )* | 0.402 | *( 0.355 - 0.454 )* | 0.321 | *( 0.157 - 0.452 )* | 0.276 | *( 0.162 - 0.421 )* |  |  |
| 24 | 0.000 | *( 0.000 - 0.000 )* | 0.394 | *( 0.346 - 0.444 )* | 0.330 | *( 0.130 - 0.481 )* | 0.275 | *( 0.147 - 0.446 )* | 0.000 | *( 0.000 - 0.000 )* | 0.404 | *( 0.353 - 0.452 )* | 0.285 | *( 0.106 - 0.452 )* | 0.311 | *( 0.169 - 0.468 )* |  |  |
| 25 | 0.000 | *( 0.000 - 0.000 )* | 0.400 | *( 0.346 - 0.451 )* | 0.289 | *( 0.080 - 0.478 )* | 0.311 | *( 0.150 - 0.487 )* | 0.000 | *( 0.000 - 0.000 )* | 0.410 | *( 0.354 - 0.458 )* | 0.243 | *( 0.064 - 0.451 )* | 0.348 | *( 0.171 - 0.503 )* |  |  |

Supplemental Table S2. Year-specific estimates in the proportions of female Steller sea lions aged 12 (peak pupping age) that were With-Pup, With-Juvenile or had No-Dependent for two natal regions in Southeast Alaska, 2005–2019. 95% CI are in parentheses; data are plotted in Figure 5. Natal regions were South (Forrester and Hazy) and North (White Sisters and Graves Rocks, Figure 1).

| Year | With-Pup | | With-Juvenile | | No-Dependent | |
| --- | --- | --- | --- | --- | --- | --- |
| North |  |  |  |  |  |  |
| <2011 | 0.592 | *( 0.513 - 0.668 )* | 0.310 | *( 0.237 - 0.380 )* | 0.096 | *( 0.062 - 0.149 )* |
| 2011 | 0.669 | *( 0.567 - 0.756 )* | 0.239 | *( 0.161 - 0.324 )* | 0.089 | *( 0.057 - 0.145 )* |
| 2012 | 0.536 | *( 0.460 - 0.616 )* | 0.361 | *( 0.283 - 0.431 )* | 0.100 | *( 0.065 - 0.156 )* |
| 2013 | 0.533 | *( 0.457 - 0.614 )* | 0.363 | *( 0.285 - 0.433 )* | 0.101 | *( 0.065 - 0.157 )* |
| 2014 | 0.756 | *( 0.644 - 0.838 )* | 0.159 | *( 0.089 - 0.254 )* | 0.082 | *( 0.050 - 0.142 )* |
| 2015 | 0.564 | *( 0.485 - 0.642 )* | 0.335 | *( 0.261 - 0.406 )* | 0.098 | *( 0.063 - 0.152 )* |
| 2016 | 0.518 | *( 0.446 - 0.595 )* | 0.377 | *( 0.302 - 0.445 )* | 0.102 | *( 0.066 - 0.158 )* |
| 2017 | 0.529 | *( 0.448 - 0.615 )* | 0.367 | *( 0.284 - 0.443 )* | 0.101 | *( 0.066 - 0.157 )* |
| 2018 | 0.465 | *( 0.400 - 0.542 )* | 0.426 | *( 0.347 - 0.491 )* | 0.106 | *( 0.068 - 0.166 )* |
| 2019 | 0.522 | *( 0.437 - 0.624 )* | 0.373 | *( 0.274 - 0.452 )* | 0.101 | *( 0.066 - 0.159 )* |
|  |  |  |  |  |  |  |
| South |  |  |  |  |  |  |
| <2011 | 0.643 | *( 0.572 - 0.704 )* | 0.261 | *( 0.207 - 0.319 )* | 0.091 | *( 0.059 - 0.144 )* |
| 2011 | 0.719 | *( 0.628 - 0.791 )* | 0.192 | *( 0.130 - 0.262 )* | 0.085 | *( 0.053 - 0.141 )* |
| 2012 | 0.584 | *( 0.506 - 0.656 )* | 0.316 | *( 0.250 - 0.383 )* | 0.096 | *( 0.062 - 0.149 )* |
| 2013 | 0.581 | *( 0.503 - 0.656 )* | 0.319 | *( 0.249 - 0.385 )* | 0.096 | *( 0.062 - 0.150 )* |
| 2014 | 0.796 | *( 0.693 - 0.866 )* | 0.122 | *( 0.067 - 0.201 )* | 0.078 | *( 0.045 - 0.140 )* |
| 2015 | 0.614 | *( 0.532 - 0.690 )* | 0.288 | *( 0.219 - 0.357 )* | 0.093 | *( 0.060 - 0.147 )* |
| 2016 | 0.564 | *( 0.483 - 0.642 )* | 0.334 | *( 0.261 - 0.404 )* | 0.097 | *( 0.063 - 0.152 )* |
| 2017 | 0.576 | *( 0.485 - 0.666 )* | 0.323 | *( 0.237 - 0.404 )* | 0.096 | *( 0.063 - 0.151 )* |
| 2018 | 0.501 | *( 0.426 - 0.587 )* | 0.391 | *( 0.308 - 0.463 )* | 0.103 | *( 0.066 - 0.160 )* |
| 2019 | 0.568 | *( 0.469 - 0.676 )* | 0.330 | *( 0.228 - 0.417 )* | 0.097 | *( 0.063 - 0.152 )* |

Supplemental Table S3. Survival estimates of Steller sea lion females in Southeast Alaska (2001–2019) by age, year and natal region or rookery. Estimates were from model 79, Appendix 4. Natal areas were South (Forrester and Hazy) and North (White Sisters and Graves Rocks, Figure 1). 95% CI are in parentheses. Survival estimates are annual probabilities (Age= 0 is survival from age 0 to age 1, ages 3–15 = constant annual survival for ages 3 to 15). For the age category 3–15 (prime-aged females), survival varied by year and was reduced during the Pacific Marine Heatwave in 2014–2016 (Hastings et al. 2023).

| Age (Yrs) | South | | White Sisters | | Graves Rocks | |
| --- | --- | --- | --- | --- | --- | --- |
| 0 | 0.578 | *( 0.538 - 0.618 )* | 0.656 | *( 0.598 - 0.710 )* | 0.715 | *( 0.616 - 0.798 )* |
| 1 | 0.712 | *( 0.657 - 0.761 )* | 0.775 | *( 0.716 - 0.824 )* | 0.819 | *( 0.738 - 0.882 )* |
| 2 | 0.894 | *( 0.845 - 0.930 )* | 0.921 | *( 0.879 - 0.950 )* | 0.939 | *( 0.895 - 0.966 )* |
| 3-15, other years | 0.936 | *( 0.926 - 0.944 )* | 0.953 | *( 0.940 - 0.963 )* | 0.964 | *( 0.944 - 0.977 )* |
| 3-15, 2014–2015 | 0.877 | *( 0.833 - 0.912 )* | 0.908 | *( 0.869 - 0.937 )* | 0.929 | *( 0.885 - 0.958 )* |
| 3-15, 2016 | 0.796 | *( 0.707 - 0.863 )* | 0.845 | *( 0.764 - 0.901 )* | 0.878 | *( 0.790 - 0.931 )* |
| 16-17 | 0.881 | *( 0.799 - 0.934 )* | 0.912 | *( 0.841 - 0.953 )* | 0.931 | *( 0.861 - 0.968 )* |
| 18+ | 0.785 | *( 0.703 - 0.851 )* | 0.836 | *( 0.758 - 0.894 )* | 0.870 | *( 0.782 - 0.927 )* |
